# Factors associated with the completeness of information provided in adverse drug reaction reports of physicians, pharmacists and consumers from Germany

**DOI:** 10.1038/s41598-025-07973-9

**Published:** 2025-07-03

**Authors:** Diana Dubrall, Patrick Christ, Severin Domgörgen, Matthias Schmid, Bernhardt Sachs

**Affiliations:** 1 Institute for Medical Biometry, Informatics and Epidemiology, University Hospital Bonn, Venusberg-Campus 1, 53127 Bonn, Germany; 2 Research Division, Federal Institute for Drugs and Medical Devices (BfArM), Kurt-Georg-Kiesinger-Allee 3, 53175 Bonn, Germany; 3 Department for Dermatology and Allergy, University Hospital RWTH Aachen, Morillenhang 27, 52074 Aachen, Germany

**Keywords:** Adverse drug reactions, Completeness of documentation, Spontaneous reporting database, Spontaneous reports, Reporter types, Epidemiology, Health care

## Abstract

The aim of this study was to identify factors associated with the completeness of spontaneous reports of adverse drug reactions (ADR) from different reporter types. We analysed 69,976 spontaneous ADR reports from physicians, 42,396 from pharmacists and 121,144 from consumers. The adjusted vigiGrade completeness score for ADR reports from EudraVigilance was used to evaluate the completeness of information provided in the structured format of each ADR report. Mean values of the vigiGrade completeness scores were calculated for various factors such as age, sex, receive year, seriousness and causal relationship. For all three reporters, the completeness of the ADR reports referring to older patients and children/ adolescents was lower than for middle-aged patients. Furthermore, generally poorly documented reports often included more than one suspected/interacting drug or no information on the patients’ sex. Additionally, ADR reports with an at least possible causal relationship were more completely documented than those with a not assessable causal relationship in a subset of 479 ADR individually assessed reports. Differences between the reporters were only observed regarding seriousness criteria and receive year. The missing of certain information parameters in ADR reports from all three reporter types may serve as indicators for a poorer overall completeness of documentation.

## Introduction

Spontaneous reporting databases are used in pharmacovigilance practice as one tool to investigate adverse drug reactions (ADRs) after marketing authorization^1^. Nowadays, ADR reports can be submitted by Health Care Professionals (HCPs) such as physicians and pharmacists or by Non-Health Care Professionals (non-HCPs) like patients and their relatives^2^. In particular, the number of ADR reports from non-HCPs, in the following designated as consumers, has increased in recent years^3,4^.

The value of ADR reports, for example in the context of dedicated pharmacovigilance procedures, depends largely on the amount and quality of information provided in each ADR report^5,6^. In case of insufficient information regarding clinically relevant data the assessment of the causal relationship between the reported ADR and the suspected drug is difficult or even not feasible. Thus, some regulatory agencies already indicated the need for tools to assess the quality of ADR reports^7,8^.

In the beginning of ADR reporting by consumers it was discussed and analysed whether these ADR reports differ from those reported by HCP, whether they can contribute to signal generation and can be considered as valuable as those from HCPs^9–12^. Among other things, it was observed that the types of drugs and ADRs reported by consumers differed from those reported by HCPs^3,9,13^. This led to the assumption that ADR reports from consumers complement ADR reports from physicians, thereby leading to a more complete picture regarding drug safety analyses^9–14^.

The quality of ADR reports in terms of the completeness of information provided in the structured format of ADR reports^8,12,15,16^ or by individual case assessments^17^ has already been examined in a few studies. One tool to measure the completeness of the information provided in the structured format of the ADR reports is the vigiGrade completeness score^8^. We also conducted such a study in which we analysed the completeness of structured information provided in ADR reports from physicians, pharmacists and consumers from Germany^4^. In this study, ADR reports from consumers were comparable regarding the achieved values of the completeness scores to those from physicians and pharmacists underlining their value. However, in this study we mainly analysed if the respective information was provided or not. No further evaluation of the investigated categories was performed. Further on, most of the studies of other research groups mentioned above, mainly considered, the completeness scores for ADR reports from different reporter types, different countries, or for specific ADRs (e.g. serious versus non-serious). A comprehensive analysis of other factors such as the individual age of the patient, the different categories of seriousness criteria, the number of reported drugs, ADRs and medical histories, the causal relationship, as well as the length of free-text descriptions has not been assessed yet.

Our hypothesis was that other factors such as the age and sex of the patients, the number of ADRs, drugs and medical histories, the seriousness of the ADR reports, the receive year, the presence of any free-text description and the specific ADR-drug combinations might be associated with the completeness of the ADR reports not only in general but also for different reporter types. Identifying factors associated with the completeness of ADR reports may help to derive measures to improve the information provided in these reports in the future. Further on, we hypothesized that the completeness of the ADR reports with an at least possible causal relationship between the intake of a drug and occurrence of an ADR might be more completely documented than those with a causal relationship classified as not assessable. If so, the measurement of completeness could help in everyday pharmacovigilance practice to prioritise ADR reports with a higher completeness of documentation for the individual case assessment.

## Methods

### Definitions

ADRs (definition described elsewhere^2^) can be reported by Health Care Professionals (HCP e.g. physicians, pharmacists), who are in Germany obliged by their professional conduct code to report ADRs, or non-Health Care Professionals (non-HCP, e.g. consumers)^2,3^. Both can report ADRs online or by a specific reporting form which is the same for HCP and non-HCP^18,19^. Following the legal definition of the guideline on good pharmacovigilance practice (GVP) of the European Medicines Agency (EMA), an ADR is classified as serious if the ADR was life-threatening, led to death, congenital anomalies, hospitalization or prolongation thereof, permanent disabilities or other medically important conditions^2^.

### Eudravigilance

EudraVigilance is the ADR database of the EMA and contains all ADRs reported in the European Economic Area (EEA)^1^. In EudraVigilance ADRs are coded in accordance with MedDRA terminology^20^ and drugs are coded by the EudraVigilance medicinal product dictionary^21^. MedDRA terminology consists of five different hierarchical levels allowing detailed and rather aggregated analyses^20^. The highest level of analyses is the System Organ Class (SOC) level describing the organ system in which the ADR occurred.

### Identification of ADR reports

The same dataset as in our previous study by Christ et al.^4^ analyzing the overall completeness and information provided in ADR reports from physicians, pharmacists and consumers from Germany was used for a more deeply analysis of the investigated categories.

**Fig. 1:**
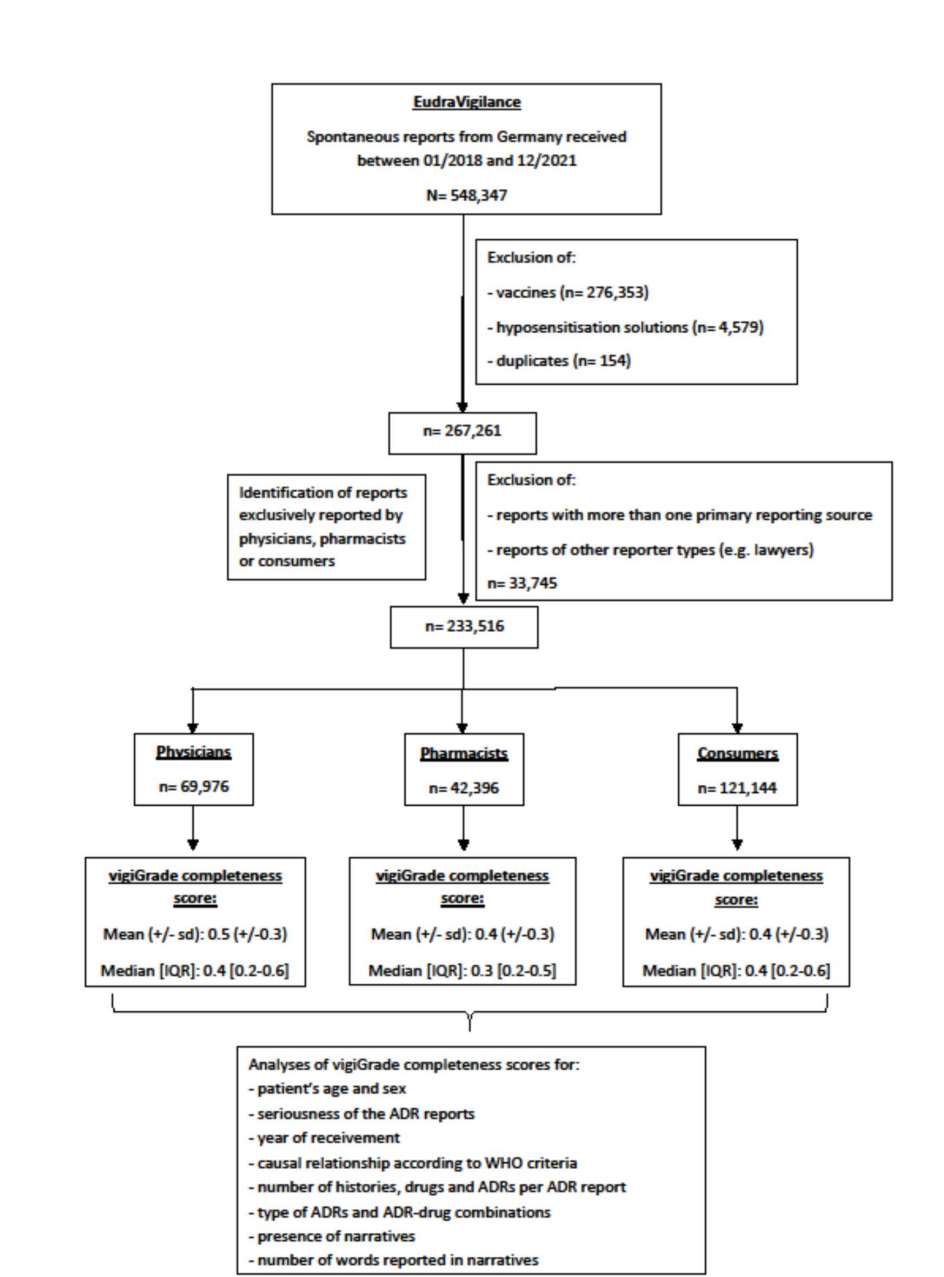
Flowchart – Identification of ADR reports and stratified analyses of their vigiGrade completeness scores.

This dataset consists of all spontaneous ADR reports from Germany received between 2018 and 2021 (n = 548,347) (Fig. 1) excluding duplicates and ADR reports in which vaccines and hyposensitization solutions were reported as suspected/interacting (remaining n = 267,261). Subsequently, all ADR reports that were exclusively reported by physicians (n = 69,976), pharmacists (n = 42,396) or consumers (n = 121,144) were used for further analysis (total n = 233,516). As the causal relationship between the suspected interacting drug/s and the reported ADR/s is not performed regularly for each ADR report in everyday pharmacovigilance practice, this information cannot be found in EudraVigilance. Thus, the causal relationship has to be assessed individually by an assessor. Since the dataset of n = 233,516 reports was too large to perform an individual case assessment, we used a random sample of already assessed ADR reports within the analysed dataset received between 2018 and 2020 originally evaluated for another project (further description see below).

Fig. 1 shows the identification of the ADR reports and their stratified analyses of vigiGrade completeness scores exclusively reported by physicians, pharmacists and consumers from Germany received between 01/2018 and 12/2021 in the ADR database EudraVigilance.

### Assessment of causal relationship of ADR reports

A random sample of 479 ADR reports from HCPs and non-HCPs received between 2018 and 2020 which were already assessed regarding their causal relationship in one of our previous projects—but are also included in the dataset of the presented analysis—was used. This subproject focused on the manual assessment of causality in a randomly drawn set of spontaneous reports. The purpose of the subproject was to generate a random set of evaluated reports to reuse these assessments in analysis like this. In this explorative study no sample size calculation was performed. The causal relationship between the reported ADR(s) and the intake of the suspected drug(s) was assessed by using the WHO criteria^22^. The causal relationship was certain in 2 (0.4%), probable/likely in 38 (7.9%), possible in 234 (48.9%), unlikely in 1 (0.2%) and not assessable in 204 (42.8%) ADR reports. 141 (29.4%) ADR reports within this sample originated from physicians, 88 (18.4%) from pharmacists and 250 (52.2%) from consumers.

### Measurement of vigiGrade completeness scores of ADR reports

The completeness of the ADR reports was evaluated by applying the vigiGrade completeness score developed by the Uppsala Monitoring Centre of the World Health Organization (WHO) for the application in VigiBase, the ADR database managed by the WHO^8^. Therefore, slight adaptions of the vigiGrade completeness score to the structure of ADR reports from EudraVigilance had to be performed^4^. The vigiGrade completeness score can be used to determine the completeness of ADR reports from each reporter type. Within this score, the presence or absence of 10 different categories of relevant information provided in ADR reports is determined. These are the report type (e.g. spontaneous report, report from study), the origin of the report (country), the primary reporting source (e.g. physician, pharmacist, consumer), the age and sex of the patient, the indication of drug therapy, the dose of drug therapy, the time to onset between the occurrence of an ADR and the start of drug therapy, the outcome of the ADR/s and the presence of free-text information (narrative). If the respective information is provided the category receives a value of 1.00. Thus, a completely documented ADR report holds a value of 1.00, meaning that information on all aforementioned categories is available. However, if the respective information is not provided, different penalties dependent on the relevance of the respective categories are applied (for a detailed description of all penalties see Supplement 1). For example, if the sex of the patient is missing, a penalty of 30% is given leading to a value of 0.70 (= 1.00–0.30) for patient’s sex. In the scoring algorithm, the values of all categories are multiplied to give the value of the completeness score of each ADR-drug combination reported in an ADR report. Finally, the completeness score of the whole ADR report is calculated by the mean of all values received per ADR-drug combination. For the vigiGrade completeness score values between 1.00 and 0.07 can be achieved. According to Bergvall et al.^8^ a report with a value ≥ 0.8 is classified as well-documented.

### Analyses of potentially associated factors on the completeness of ADR reports

For all three reporter types several factors that might be associated with the completeness of the ADR reports were investigated. In case of documentation there are some characteristics classified as important for a ‘well-documented’ ADR report. Among others, these are patient-related data (e.g. demographics, medical history), drug-related data (e.g. dose) and ADR-related data (e.g. time to onset, seriousness). Since some of the information such as the course of an ADR are only reported in more detail in the free-text descriptions (narratives), the length of the narratives (measured by the number of words) may also be one important characteristic. Further on, since ADR reporting became more popular, the completeness of ADR reporting may also have been increased in the analysed period. Finally, we wanted to verify the hypothesis that ADR reports with at least possible causal relationship achieve a higher vigiGrade completeness score than those classified as not assessable. Therefore, the following factors with their subgroups were analysed:

- age of the patient: the individual age of the patient in years,
- sex of the patient: female, male and unknown,
- seriousness and its categories: yes, no and not available,
- receive year: 2018, 2019, 2020 and 2021,
- causal relationship: certain, probable/likely, possible, unlikely, unclassifiable, unassessable,
- presence of free-text information (= narratives): yes, no,
- number of words included in narratives,
- reported ADRs on the SOC level of MedDRA terminology^13^,
- number of reported suspected/interacting drugs, histories and ADRs.

Regarding the number of words in narratives, the analysed datasets only include the narratives of the first 3000 characters. For the evaluation of the association between the number of words used in narratives and the values of the vigiGrade completeness scores of the reports, reports with more than 3000 characters and reports without any free-text description were excluded. We also analysed the number of words contained in our random sample of ADR reports with a certain, probable, possible, unlikely and not assessable causal relationship.

Concerning the reported ADR-drug combinations, the 25 most frequently reported ADR-drug combinations for the ADR reports of all three reporter types were determined.

To investigate the reasons for differences of the mean values of the vigiGrade completeness scores between some of the categories, more detailed analyses of information provided in each ADR report were performed. Therefore, the age groups 0–20 years, 21–59 years and 60 years and older were formed for the more detailed analyses of information provided in ADR reports dependent on patient’s age.

### Calculation of the mean values of the vigiGrade completeness scores per subgroup and statistical analyses

In each subgroup the mean and median values of the vigiGrade completeness scores and their standard deviations and interquartile ranges were calculated, respectively.

Concerning numerical variables, linear regression lines and nonlinear regression lines estimated by locally weighted scatterplot smoothing (loess) were fitted to the points of the scatterplots and their 95.0% confidence intervals (CI) were determined. Thereby the analysed categories were considered as independent variables and the mean values of the vigiGrade completeness scores as dependent variables. For the numerical variables, a threshold of at least three ADR reports per category was set for reports to be included in the analyses to reduce the impact of extreme values from individual reports.

Forcategorical variables, the differences between the mean values of the vigiGrade completeness scores in the subgroups were analysed by using an unpaired t-test with Holm’s correction for multiple testing. Thereby the analysed subgroups were considered as independent variables and the mean values of the vigiGrade completeness scores as dependent variables. Concerning the mean values of the vigiGrade completeness scores in the subgroups defined by the causal relationship of the ADR report, the subgroups certain (n = 2) and unlikely (n = 1) were excluded from statistical analysis due to their small number of reports.

For all analyses R version 4.3.2 was used^23^.

## Results

### Age of the patients

**Fig. 2:**
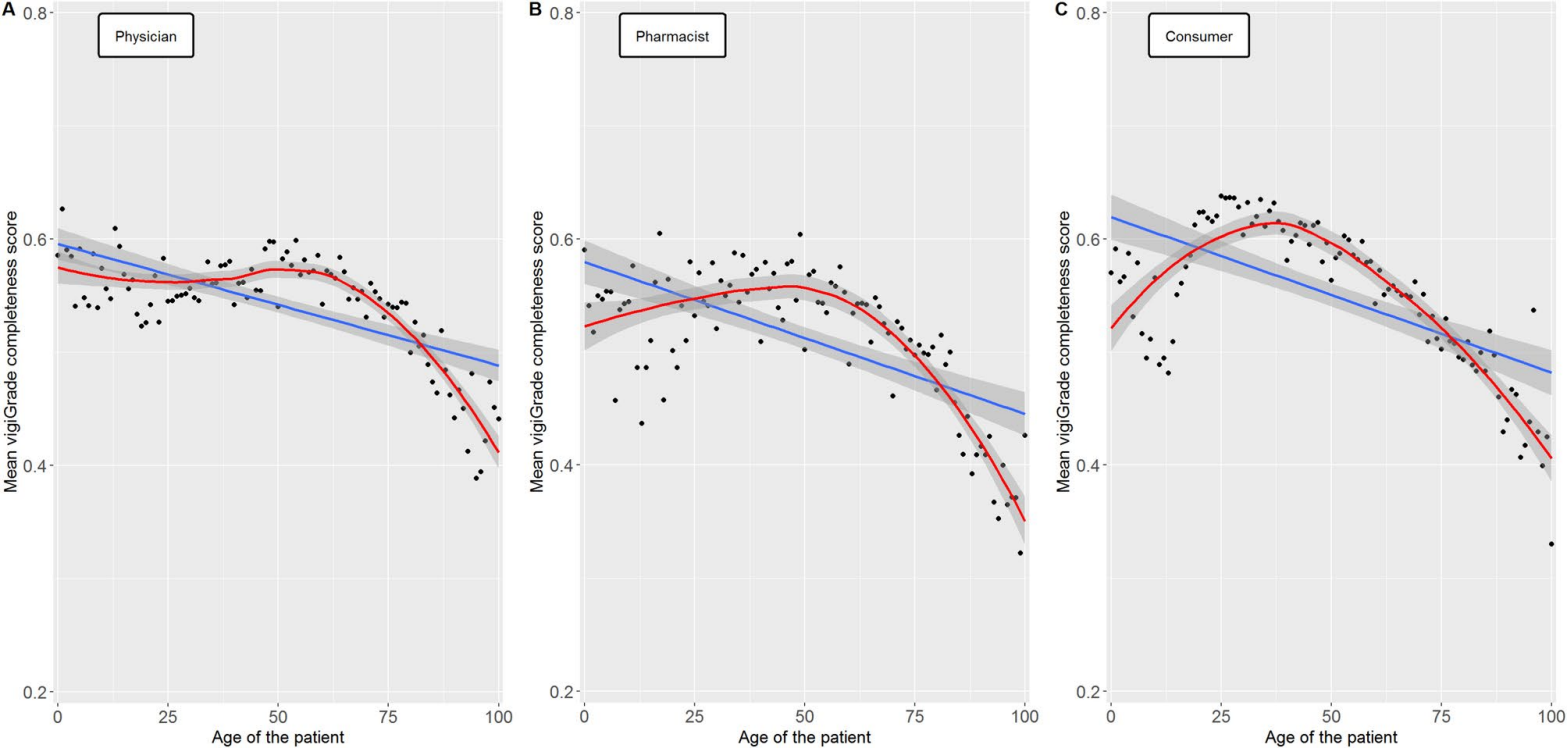
Association of patient’s age on the completeness of ADR reports.

In ADR reports from all three reporter types, the completeness of information provided was lower if patients were older than 60 years (Fig. 2). Whereas in reports from consumers and pharmacists the highest mean values of the completeness scores were achieved for patients aged 21–60 years, the mean values for patients aged 0–20 years tended to be lower. The latter was to a lower extent observed for ADR reports from physicians in which the values of the completeness scores for patients aged 0–60 were rather similar.

Further analyses showed that especially the information time to onset, indication and dose of drug therapy and the outcome of the ADR were less often reported in ADR reports referring to patients 60 years and older, compared to patients younger than 60 years (Supplement 2).

Fig. 2 shows the mean values of the vigiGrade completeness scores depending on the patient’s age in reports from physicians, pharmacists and consumers. The blue line represents the linear regression line and the red line the loess regression line. For smoothing of the latter, a span of 0.90 was set for ADR reports from all three reporter types. The gray borders represent the 95.0% confidence intervals.

### Sex of the patients

**Fig. 3:**
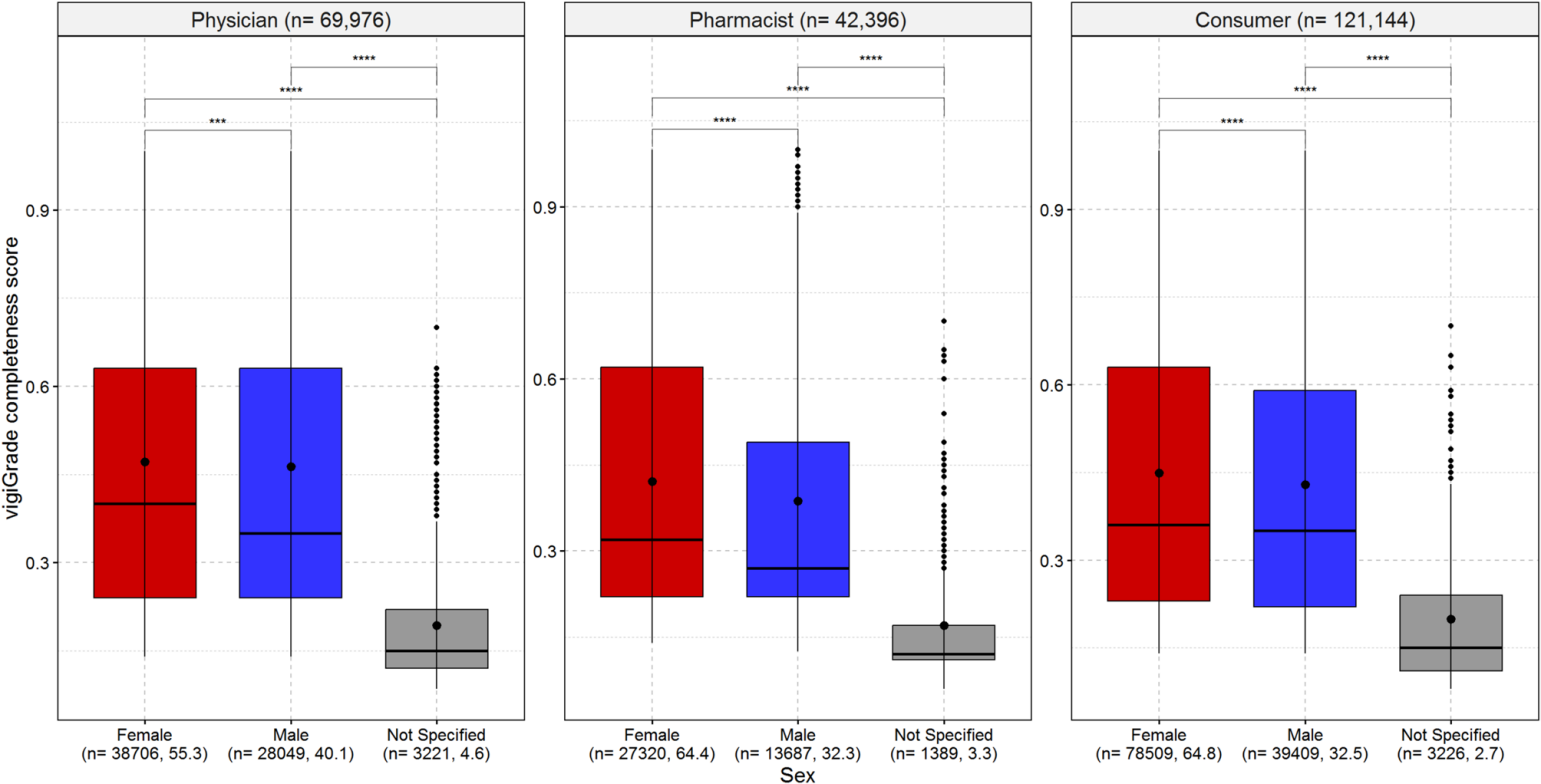
Association of patient’s sex on the completeness of ADR reports. p-values coded as: 1–0.05 ‘ns’; > 0.05–0.01 ‘*’; > 0.01–0.001 ‘**’; > 0.001–0.0001 ‘***’; > 0.0001–0 ‘****’.

Reports referring to females were more completely documented than reports referring to males for all three reporter types (Fig. 3). In addition, if the sex of the patient was reported as not specified, this was associated with rather poorly documented reports. More detailed analyses showed that the time to onset, the outcome of the ADR, the indication and dose of drug therapy were in proportion slightly more often reported in ADR reports referring to females than to males (Supplement 2).

Fig. 3 shows the boxplots of the vigiGrade completeness scores with the corresponding mean and median values depending on patient’s sex in reports from physicians, pharmacist and consumers. An unpaired t-test with Holm’s correction for multiple testing was performed to analyse differences between the mean values of the vigiGrade completeness scores of the analysed categories.

### Seriousness criteria of the ADR reports

Reports classified as serious reported from physicians were slightly better documented than those classified as non-serious (Supplement 3). The opposite was observed for ADR reports from pharmacists, whereas the seriousness of the ADR reports showed no association with the completeness of ADR reports from consumers.

Considering the individual seriousness criteria (see 2.1), reports from physicians coded with “yes” concerning the criteria death, hospitalization, life-threatening and disabling achieved higher mean values of the vigiGrade completeness scores than those coded with “no”. This was also observed for reports from pharmacists except for the criterion hospitalization and from consumers except for the criterion death.

### Receive year of the ADR reports

**Fig. 4:**
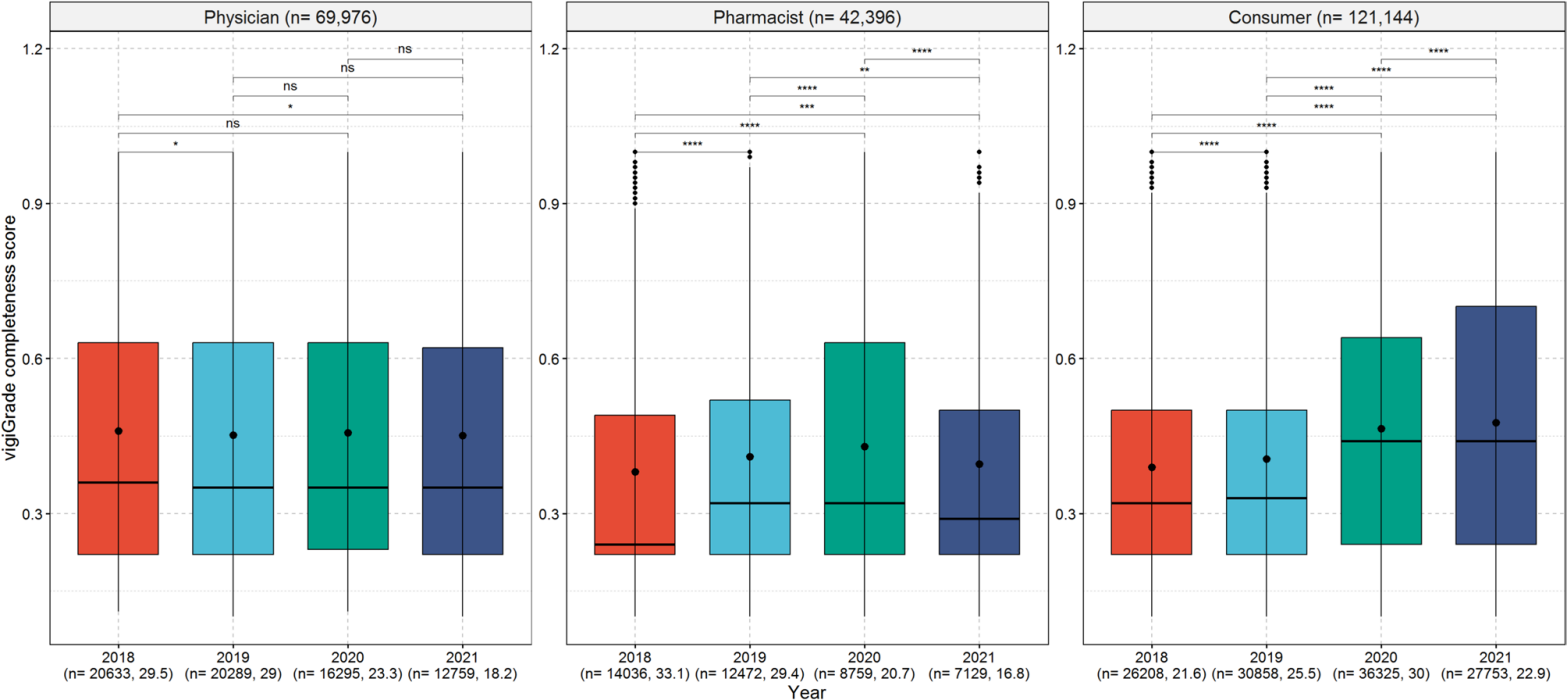
Association of the receive year of the ADR reports with the completeness of ADR reports. p-values coded as: 1–0.05 ‘ns’; > 0.05–0.01 ‘*’; > 0.01–0.001 ‘**’; > 0.001–0.0001 ‘***’; > 0.0001–0 ‘****’.

Over the years, the mean values of the vigiGrade completeness scores for the reports from physicians were consistent (Fig. 4). In contrast, in reports from pharmacists the mean values of the vigiGrade completeness scores increased from 2018 to 2020 and declined from 2020 to 2021, while the mean values of the vigiGrade completeness scores in reports from consumers increased from 2018 to 2021. Further analyses revealed that especially information concerning the time to onset, the outcome of the ADR and to a lesser extent the indication of drug therapy were more often provided in ADR reports from consumers received in 2020 and 2021 compared to those received in 2018 and 2019. (Supplement 2, Table 4). In contrast, information regarding these three categories was in proportion less often reported in ADR reports from pharmacist received in 2021 compared to the previous years. Information concerning the age of the patient was proportionally more often provided in ADR reports from pharmacists in 2021 than before.

Fig. 4 shows the boxplots of the vigiGrade completeness scores with the corresponding mean and median values depending on the receive year of the ADR reports from physicians, pharmacists and consumers. An unpaired t-test with Holm’s correction for multiple testing was performed to analyse differences between the mean values of the vigiGrade completeness scores of the analysed categories.

### Number of histories per ADR report

For all three reporter types the mean values of the vigiGrade completeness scores were lower if only one history of the patient was reported (Supplement 4) compared to two to four histories in ADR reports from physicians and pharmacists or up to ten histories in ADR reports from consumers. Accordingly, the mean values of the vigiGrade completeness scores were higher or lower in ADR reports from physicians and pharmacists with more than five patient’s histories and ADR reports from consumer with more than eleven patient’s histories.

### Number of drugs per ADR report

The highest mean value of the vigiGrade completeness scores was achieved if only one drug was reported as suspected/interacting in ADR reports from all three reporter types (Fig. 7). The more drugs have been reported as suspected/interacting per ADR report, the lower the mean values of the vigiGrade completeness score which were calculated.

**Fig. 5:**
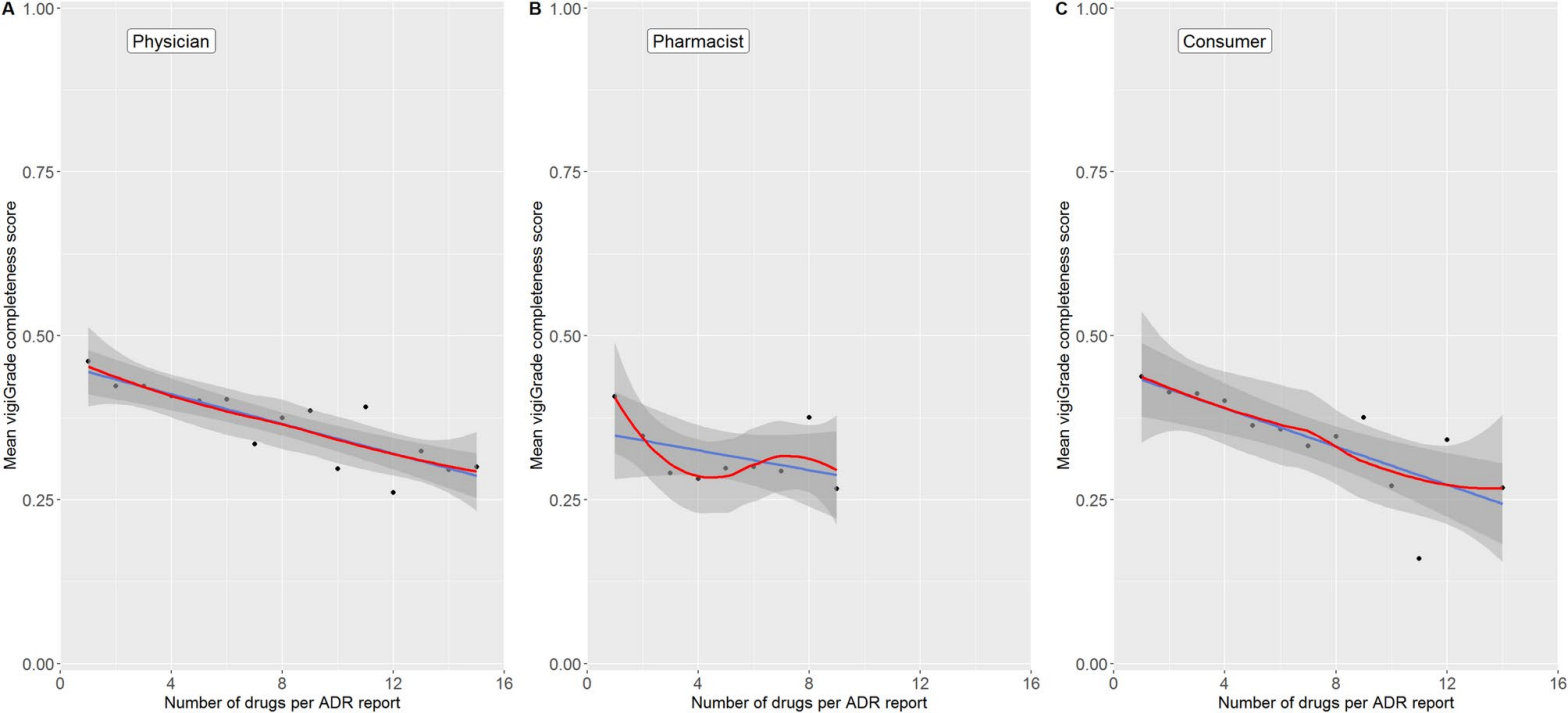
Association of the number of drugs reported as suspected/interacting in each ADR report with the completeness of the ADR report.

Fig. 5 shows the mean values of the vigiGrade completeness scores depending on the number of drugs reported as suspected/interacting in ADR reports from physicians, pharmacists and consumers. The blue line represents the regression line and the red line the loess regression line. For smoothing of the latter, spans of 0.90, 0.90 and 0.95 were set for ADR reports from physicians, pharmacists and consumers, respectively. The gray borders represent the 95.0% confidence intervals.

### Number of ADRs per ADR report

In ADR reports from all three reporter types, the mean values of the vigiGrade completeness scores increased from reports including solely one ADR to those including up to seven ADRs per ADR report (Supplement 5). A further increase was observed for ADR reports from consumers for reports in which up to 18 ADRs were reported. In ADR reports from physicians and pharmacists the trends of the mean values of the vigiGrade completeness scores for reports with more than 7 ADRs per ADR report were inconsistent. The same applies to ADR reports from consumers with more than 18 ADRs per ADR report.

### Type of ADRs on the SOC level

For ADR reports from physicians, the highest mean values of the vigiGrade completeness scores were achieved in ADR reports describing ADRs occurring in the SOCs ear and labyrinth disorders (0.53 (± 0.28)), hepatobiliary disorders (0.53 (± 0.27)) and musculoskeletal and connective tissue disorders (0.53 (± 0.27)) (Supplement 6). In contrast, the lowest mean values for ADR reports from physicians were calculated for ADR reports referring to injuries, poisoning and procedural complications (0.41 (± 0.22)), investigations (0.43 (± 0.25)) and congenital, familial and genetic disorders (0.43 (± 0.23)).

Compared to that, reports from pharmacists related to the SOCs social circumstances (0.57 (± 0.28)), ear and labyrinth disorders (0.54 (± 0.30)) and hepatobiliary disorders (0.49 (± 0.30)) achieved the highest mean values and those related to the SOCs congenital, familial and genetic disorders (0.26 (± 0.09)), surgical and medical procedures (0.29 (± 0.21)) and injury, poisoning and procedural complications (0–31 (± 0.20)) the lowest mean values.

While for ADR reports from consumers those containing ADRs occurring in the SOCs social circumstances (0.51 (± 0.26)), cardiac disorders (0.49 (± 0.26)) and pregnancy, puerperium and perinatal conditions (0.49 (± 0,22)) received the highest values, those occurring in the SOCs product issues (0.31 (± 0.19)), surgical and medical procedures (0.36 (± 0.21)) and neoplasms benign, malignant and unspecified (incl. cysts and polyps) (0.37 (± 0.21)) received the lowest values.

Notably, the mean values of the vigiGrade completeness scores for all 27 SOCs fluctuated less strongly for ADR reports from physicians (maximum = 0.53, minimum = 0.41), than for ADR reports from consumers (maximum = 0.51, minimum = 0.31) and pharmacists (maximum = 0.57, minimum = 0.26).

### Type of ADR-drug combinations reported

Considering the 25 most frequently reported ADR-drug combinations from all three reporter types, especially drug or product related problems such as drug ineffective – levonorgestrel in reports from physicians, device breakage – ethinylestradiol in report from pharmacists and product leakage – insulin in reports from consumers seemed to be less completely documented (Supplement 7). The same applied for well-known ADR-drug combinations (e.g. haematochezia – acetylsalicylic acid) or ADR-drug combinations, which may be confounded by indication (multiple sclerosis relapse – natalizumab) in ADR reports from physicians. However, myalgias related to statins received the highest values of the vigiGrade completeness score considering the 25 most frequently reported ADR-drug combinations from physicians. Compared to that, several ADR-drug combinations describing injection site reactions achieved the highest values in the ranking of the top 25 ADR-drug combinations from consumer reports. Notably, ADR-drug combinations related to the drug minoxidil and ADRs describing application site reactions received the highest values in the ADR reports from pharmacists.

### Causal relationship of the ADR report

**Fig. 6:**
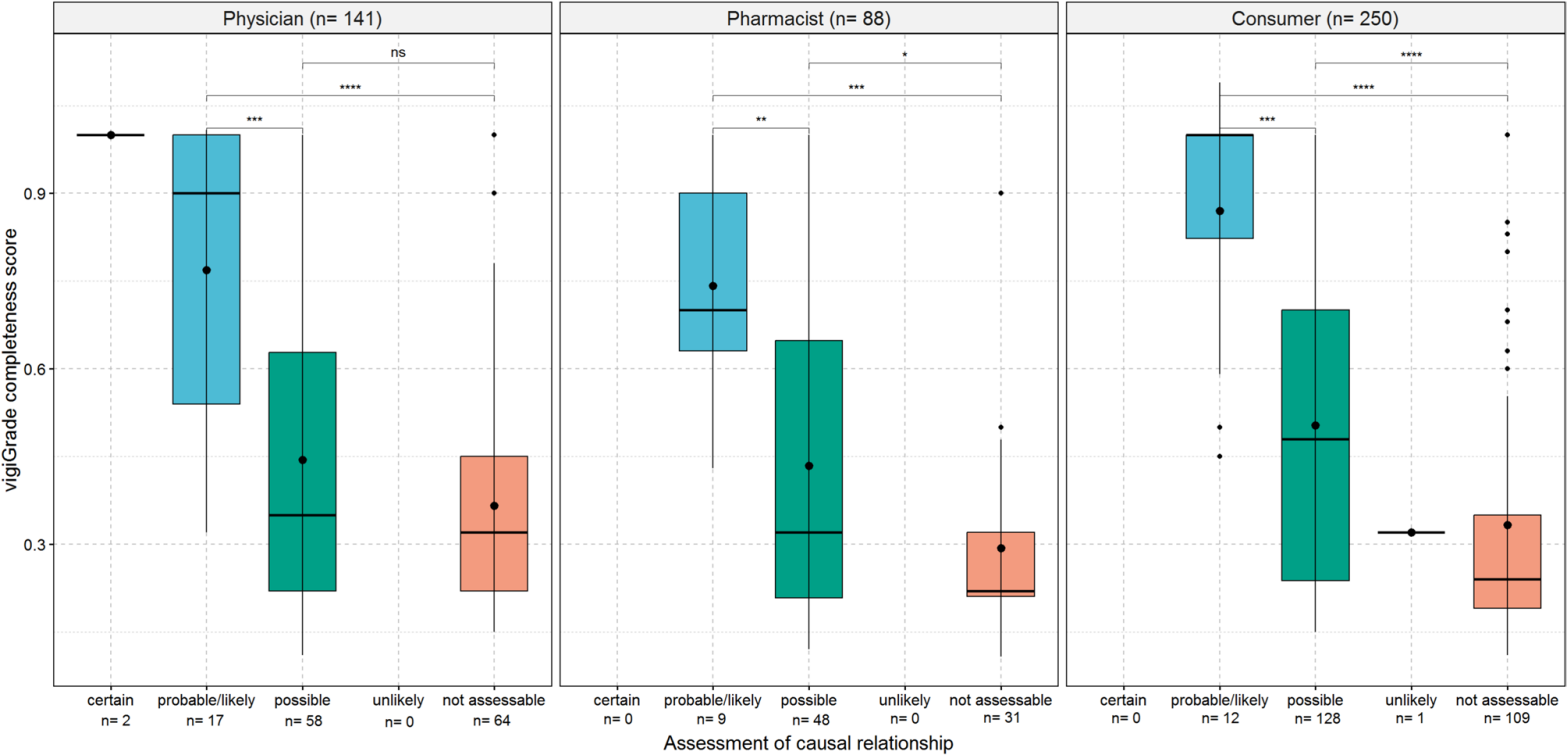
Association of the causal relationship of the ADR report with the completeness of the ADR report. p-values coded as: 1–0.05 ‘ns’; > 0.05–0.01 ‘*’; > 0.01–0.001 ‘**’; > 0.001–0.0001 ‘***’; > 0.0001–0 ‘****’.

For all three reporter types, the mean values of the vigiGrade completeness scores were higher for ADR reports in which the causal relationship was assessed as at least possible (Fig. 6). Even if the results of the t-test between the categories possible and not assessable were not that obviously different in ADR reports from physicians as in ADR reports from pharmacists and consumers, the numerical values of the mean and median vigiGrade completeness scores were noticeably lower for ADR reports classified as not assessable compared to those with a possible causal relationship.

Fig. 6 shows the boxplots of the vigiGrade completeness scores with the corresponding mean and median values depending on the causal relationship of the ADR reports from physicians, pharmacists and consumers. An unpaired t-test with Holm’s correction for multiple testing was performed to analyse differences between the mean values of the vigiGrade completeness scores of the analysed categories. The categories certain and unlikely could not be included in statistical analysis due to their low number of reports.

### Presence of narrative and number of words used

**Fig. 7:**
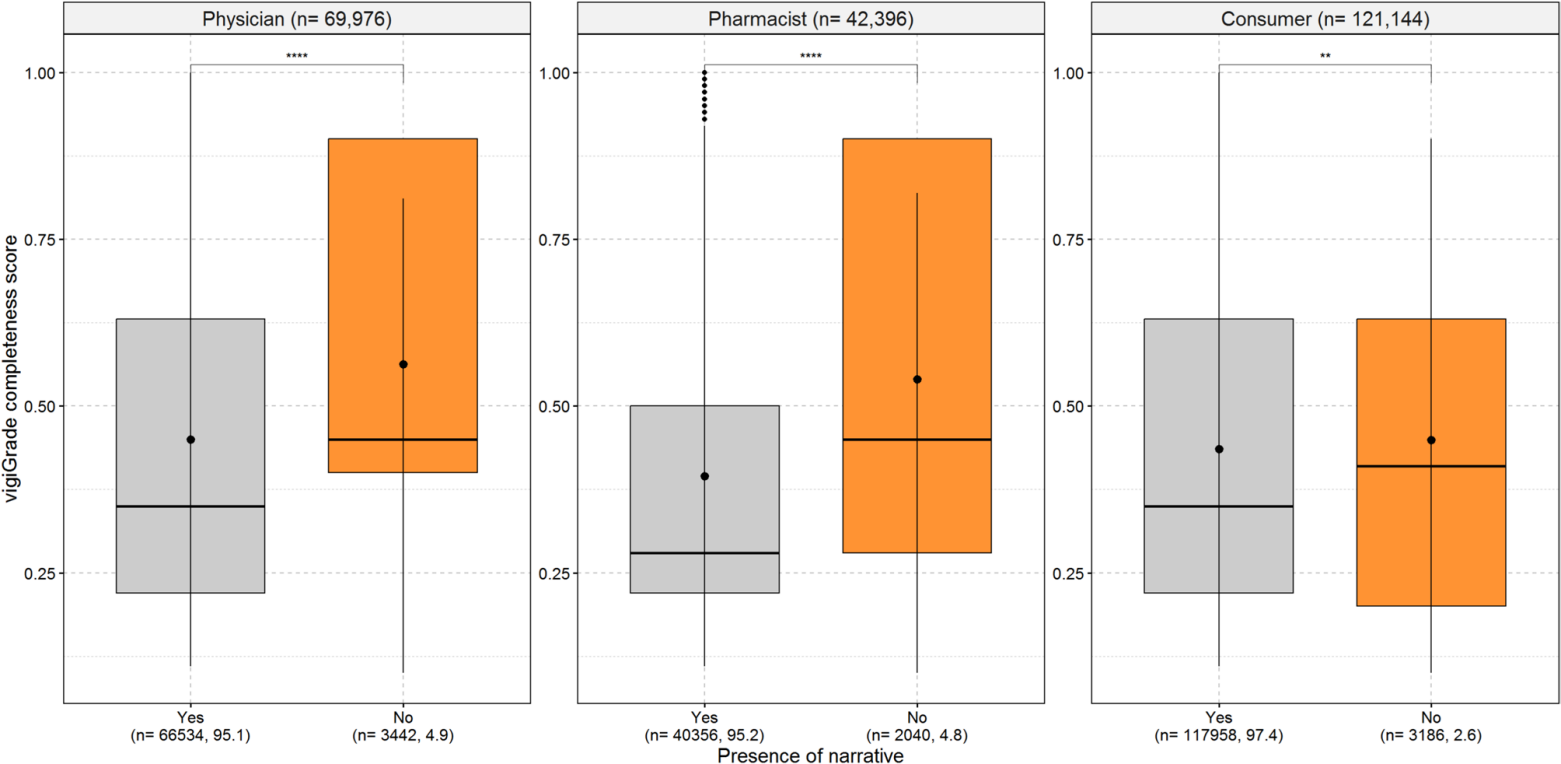
Association of presence of narratives with the completeness of the ADR report. p-values coded as: 1–0.05 ‘ns’; > 0.05–0.01 ‘*’; > 0.01–0.001 ‘**’; > 0.001–0.0001 ‘***’; > 0.0001–0 ‘****’.

**Fig. 8:**
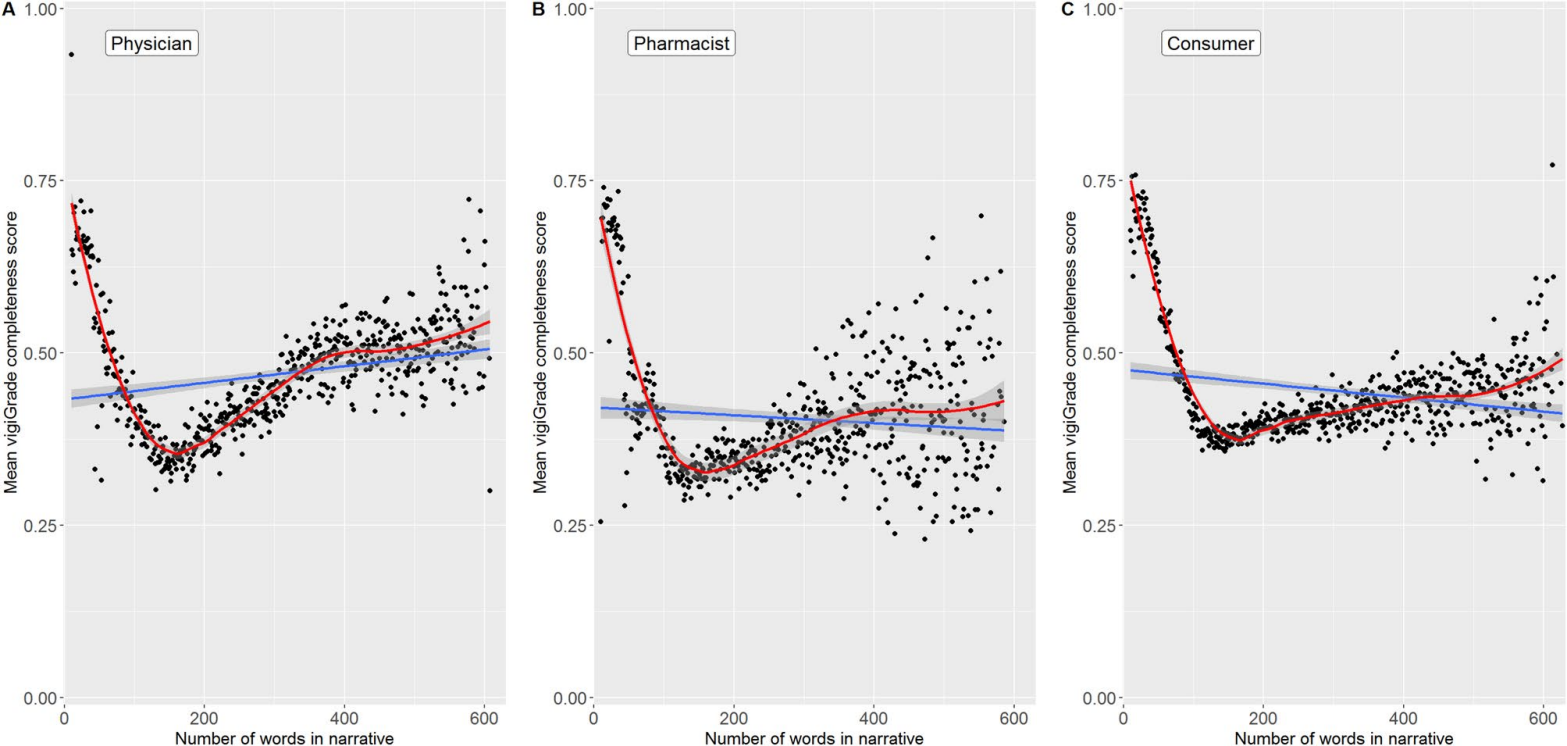
Association of the number of words reported in narratives with the completeness of ADR reports.

Even though this only concerns a small proportion of reports, we observed that reports without any free-text information in the narrative were more completely documented regarding the structured information than those with free-text information (Fig. 7). This applied to all three reporter types but was less pronounced for ADR reports from consumers compared to those from physicians and pharmacists. Considering the number of words used in the narratives, reports with approximately 100 words or less received the highest mean values of the vigiGrade completeness scores (Fig. 8). In general, a downward trend of the mean values of the vigiGrade completeness scores was observed for reports within the range of 0–125 words. In reports with narratives with more than 125 words, an upward trend of the mean values of the vigiGrade completeness scores was observed again. The distribution of the mean values of the vigiGrade completeness scores was rather inconsistent for reports with approximately more than 500 words. Interestingly, in our random sample, ADR reports with a probable causal relationship contained in median a lower number of words in the narratives and ADR reports with a possible causal relationship a higher number of words compared to ADR reports, in which the causal relationship was not assessable (Supplement 8).

Fig. 7 shows the boxplots of the vigiGrade completeness scores with the corresponding mean and median values depending on the presence of narratives in the ADR reports from physicians, pharmacists and consumers. An unpaired t-test with Holm’s correction for multiple testing was performed to analyse differences between the mean values of the vigiGrade completeness scores of the analysed categories.

Fig. 8 shows the mean values of the vigiGrade completeness scores depending on the number of words used in the narrative per ADR report from physicians, pharmacists and consumers. The blue line represents the regression line and the red line the loess regression line. For smoothing the latter, a span of 0.50 was set for ADR reports from all three reporter types. The gray borders represent the 95.0% confidence intervals. The analysed datasets only include the narratives of the first 3000 characters. For the evaluation of the association between the number of words used in narratives and the vigiGrade completeness scores of the reports, reports with more than 3000 characters and reports without any free-text description were excluded.

## Discussion

In this study we performed a comprehensive analysis investigating factors associated with the completeness of ADR reports from physicians, pharmacists and consumers. Several factors such as age, and sex of the patient could be identified as associated factors. Based on our results, further measures could be initiated to improve the completeness of ADR reports.

### Demographical parameters

All three reporter types should pay increased attention to the completeness of ADRs reports referring to older adults as well as children and adolescents. The completeness of ADR reports referring to these age groups is particularly important because these patients belong to i) vulnerable patient groups, and ii) patient groups for whom data regarding drug therapy is often insufficient at the time of marketing authorisation. In our study, the higher the age of the patients, the lower the values of the completeness scores of the ADR reports which were achieved. We assume that, among other things, this might be related to the fact that older adults take more drugs than younger adults^24^. As our analysis showed, the more drugs reported as suspected/interacting, the lower the completeness of the ADR reports. Different drugs could probably be prescribed by different physicians or being handed out in several pharmacies. Thus, with respect to physicians and pharmacists not all information regarding the entire drug therapy may be known. Additionally, some of the drugs may have already been used over a long period of time, so that the respective reporter may not remember specific information such as the exact start date of drug therapy. With respect to reports from consumers, other studies described that some patients may not be fully aware about all details of their drug therapy (e.g. concerning their indication)^25^. Especially for older patients’ knowledge about their own medication seems worse than for patients younger than 65 years^26^. Literature is conflicting as to whether long-term^25^ or short-term^26^ drug therapy is associated with better medication knowledge. Also, communication problems with older patients e.g. due to dementia or hearing loss are a conceivable explanation for a lower completeness of ADR reports from physicians, pharmacists or relatives of the patient. Further on, the causal relationship between drug therapy and ADR occurrence may be unclear as certain ADRs may also reflect progression of underlying diseases or signs of aging. In addition, the higher number of drugs used may also lead to difficulties in identifying the causative drug. In summary, these uncertainties may all impact on the completeness of ADR reporting to an unknown extent.

Communication problems may also apply to younger children who may not yet be able to express themselves and their perceived ADRs precisely explaining the lower values of the completeness scores in ADR reports referring to children from pharmacists and consumers. Notably, this observation applied to a lower extent to ADR reports from physicians. Possibly, physicians take this patient group very seriously and spend more time on ADR reporting for children. Another explanation for this finding could be that the types of ADRs reported for children from physicians, pharmacists and consumers differ. Physicians may report ADRs more often which are easier for them to recognize e.g. by diagnostic and laboratory parameters as also seen in a previous analysis of ADR reports in general^3^. It could be discussed if a reminder within the ADR online reporting tool, occurring as soon as an ADR report referring to these patient groups is generated, could help to emphasize their importance and thereby increase the completeness of these reports.

Concerning the differences regarding the sexes, other studies already observed that females not only report more ADRs^27^ but may also communicate and describe symptoms in general in more detail than males^28^. In addition, medication knowledge about their own drug therapy was better for females than for males in the aforementioned studies^25,26^. Both facts may also favour a higher completeness of ADR reports referring to females than to males from all three reporter types. Our study, as well as one study from Midi-Pyrénées Regional Pharmacovigilance Center in France, showed that unknown sex is an indicator for poorly documented ADR reports^5^. In this context, we would like to point out that the sex of the patient is a very important information to identify and build further knowledge about sex-specific ADRs.

### Seriousness of the ADR reports

Physicians may not only report serious ADRs more often^29^, but also more completely than non-serious ADRs, as observed in our study and by others^5^. In contrast, serious reports from pharmacists, especially those coded with hospitalization, were clearly less completely documented than non-serious reports. Except for hospital pharmacists, this finding might reflect that pharmacists from community pharmacies are not as close to in-patients. In addition, these ADRs are probably reported with a delay. Both factors could potentially influence the completeness of these ADR reports from pharmacists as detailed information may not or no longer be known at the time of reporting. Regarding reports from consumers, it is known that for consumers also non-serious ADRs that might affect their quality of life are of importance^3^. This could explain why no differences between the completeness of serious and non-serious ADR reports were observed for reports from consumers in our study. Further studies from Australia and China, including several reporter types but mainly HCP, showed that, serious ADRs were more completely reported than mild ones or those with unknown severity^30,31^. Of course, the completeness of the ADR reports should not depend on the seriousness of the ADR. However, these ADR reports are of particular importance as they can pose a higher burden to the patient and to the healthcare system. Based on our analysis, especially pharmacists and consumers should pay more attention to report all relevant information regarding serious ADRs.

### Number of histories, ADRs and drugs reported as suspected per ADR reports

Contrary to our expectations, ADR reports with more than one ADR or history of the patient were more completely documented than ADR reports with only one ADR or history. This could indicate that reporters who create such reports put a lot of effort into ADR reporting. It could also show that reports containing only one ADR, or symptoms condensed to one diagnosis, are rather less completely documented. It was striking that the effect of a more detailed reporting was not observed if multiple ADRs (> 7) and histories (> 10) were reported per ADR report. One possible reason could be that it becomes increasingly difficult and time consuming to transmit everything in the structured format of the ADR report. Another option could be that different types of patients or other so far unknown factors play a role.

In contrast to the number of ADRs and patient’s histories per ADR report, the increasing number of drugs reported as suspected per ADR report was negatively associated with the completeness of the ADR report. As already mentioned, lack of knowledge about drug therapy^25,26^, higher complexity of reporting or difficulties in identifying the causative drugs may be possible reasons.

### Type of ADRs and ADR-drug combinations

It was striking that the range of the mean values of the vigiGrade completeness scores regarding the ADRs on the SOC-level was smaller for ADR reports from physicians compared to ADR reports from pharmacists and consumers, which could reflect their medical expertise. Once again, our analysis showed that consumers may not only report subjectively perceived ADRs more often, but also more completely since ADR reports related to the SOC social circumstances achieved the highest mean values of the vigiGrade completeness scores in their ADR reports.

Based on the completeness scores of the 25 most frequently reported ADR-drug combinations in ADR reports from physicians, pharmacists and consumers, one could deduce that especially ADR reports describing drug-related problems and medication errors were less completely reported. We want to emphasize this finding because ADRs caused by medication errors can in general be avoided and, therefore, be reduced to a minimum if they are known and communicated to physicians, pharmacists and consumers. However, it is also possible that the actual ADR reporting form^18,19^ is not optimal for the reporting of medication errors and drug-related problems, as it was designed for the reporting of ADRs. Thus, some of the information may not apply to the information categories of the structured format of these reports and may only be reported in the free-text descriptions. Because medication errors are an important issue, we investigate this topic in another project^32^.

In addition, it may be speculated that ADR reports containing ADRs to well-known drugs (e.g. acetylsalicylic acid – hematochezia) or ADRs that might also reflect progression of diseases (e.g. natalizumab –multiple sclerosis relapse) are likewise less completely reported than ADR reports describing ADRs to newer drugs.

### Receive year of the ADR report

In the past, not only the number of ADR reports from consumers increased^3,4^ but also the completeness of ADR reports from consumers, as observed in our study. Even if ADR reports to vaccines are not included in this study, this could reflect a higher awareness of ADR reporting by consumers possibly also triggered by the COVID-19 pandemic, which made ADR reporting more known to the public. In contrast, the completeness of ADR reports from pharmacists decreased in the last year of our analysis, which is why we want to motivate especially this reporter group to include as much information as possible in an ADR report.

### Causal relationship of the ADR report

Our analysis highlights that ADR reports from all three reporter types with an at least possible causal relationship are associated with higher mean values of the vigiGrade completeness scores than ADR reports in which the causal relationship was not assessable. Thus, the evaluation of the vigiGrade completeness score may be useful in pharmacovigilance practice to identify reports with higher values, which could be analysed first in the context of signal validation or when specific pharmacovigilance questions arise. Notably, the number of ADR reports for the categories certain and unlikely were too small to make reliable statements. However, it seems plausible that ADR reports classified as certain are associated with higher vigiGrade completeness score.

### Presence of narratives in ADR reports

In addition to the absence of narratives, the values of the completeness scores of the reports were highest if the narrative was rather short (< 125 words) and increased again for ADR reports with a narrative of more than 300 words. In our random sample of reports with an assessment of the causal relationship (n = 479), reports with a probable causal relationship (n = 38/479, 7.9%) tended to contain a lower number of words in the narrative than ADR reports with a possible (n = 234/479, 48.9%) or not assessable (n = 204/479, 42.8%) causal relationship. This observation applied to all three reporter types. However, the number of reports with a probable causal relationship was low (physicians: 12.1% (17/141); pharmacists: 10.2% (9/88), consumers: 4.8% (12/250)). Hence, the results should be interpreted with caution and any connection between a higher score of documentation, a lower number of words and a probable causal relationship could be due to various (also interacting) factors including chance. In case of short narratives, the person reporting may have explained everything briefly and plausibly, or certain information—which could raise any doubts regarding the causal association between the drug and the ADR—may be missing. In the latter case, brevity would imply clarity of the causal relation. Additionally, specific ADRs like anaphylactic reactions may not need to be explained in much detail within the narrative to enable a potential assessment for probable causality. In contrast, some of the reports with very long narratives may present complex cases with several suspected, interacting or concomitant drugs, various ADRs and histories of the patients. Additional information not relevant to the case may also be reported in the narratives, which then may not be included in the structured format of the ADR reports but increases the number of words reported, too. Further on, narratives with a higher number of words may also reflect reports with several follow-up reports. In some complex cases with very long narratives, it may be difficult to assess the causal relationship, but in others, it helps to clearly evaluate the causal relationship. Ultimately, definitive statements are not possible without assessing a larger sample of reports individually. This could be the subject of further investigations.

Ideally, all information relevant to the case should be included in the structured format of the ADR report. Future studies could investigate whether this is the case—especially in narratives with a higher number of words. If not, further measures could be considered to transfer information from the narratives to the structured format of the ADR reports.

### Strengths and limitations

The strength of our analysis is the detailed consideration of several factors that might be associated with the completeness of ADR reporting.

One of the major limitations is that only information provided in the structured format as well as the number of words in the narratives was considered but not the relevance or the quality of the reported information. Further on, only ADR reports from physicians, pharmacists and consumers from Germany were considered. It is unclear whether the results also apply to ADR reports from other countries. However, this could be another research question, which can be investigated in further studies.

## Conclusion

In line with the aim of the study, we could identify factors that seem to be associated with the completeness of ADR reports for all three reporters. In this respect, there is potential for improvement especially for ADR reports referring to older adults, children and adolescents and for ADR reports with more than one drug reported as suspected/interacting in ADR reports from all three reported types. The reasons that lead to a less completely reporting of some of these factors (e.g. older age of the patients) need to be investigated in further studies to develop targeted measures to increase the completeness of information provided in these ADR reports. Further on, the evaluation of a random sample indicated that ADR reports with an at least possible causal relationship may be more completely documented than those with a not assessable causal relationship.

## Supplementary Information

Supplementary Information 1.

Supplementary Information 2.

Supplementary Information 3.

Supplementary Information 4.

Supplementary Information 5.

Supplementary Information 6.

Supplementary Information 7.

Supplementary Information 8.

## Data Availability

The datasets analysed during the current study are not publicly available due to data protection requirements. The data that support the findings of this study are available from the European Medicines Agency but restrictions apply to the availability of these data, which were used under license for the current study, and so are not publicly available. Data are however available from the Corresponding author upon reasonable request and with permission of the European Medicines Agency. A restricted dataset of the pseudonymised ADR reports from EudraVigilance is available to the public (https://www.adrreports.eu/en/search.html). The individual pseudonymised ADR reports are not publicy available. In order to fulfill their legal obligations distinct levels of access to EudraVigilance are provided for various stakeholders (https://www.ema.europa.eu/en/human-regulatory/research-development/pharmacovigilance/eudravigilance/access-eudravigilance-data). Being one of the competent authorities in Germany, the Federal Institute for Drugs and Medical Devices is granted with the highest level of access covering the individual spontaneous adverse drug reaction (ADR) reports. However, a lower level of access is granted to the public thereby enabling researchers to perform the same analysis in EudraVigilance albeit with aggregated data. For further information regarding the processing of personal data in the context of the operation of EudraVigilance Human we refer to the European Medicines Agency’s Data Protection Notice for EudraVigilance Human. Information concerning the privacy policy and data processing of the Federal Institute for Drugs and Medical Devices can be found here: https://www.bfarm.de/EN/Service/Privacy-policy/_node.html.
