# Supplementary material for: Factors associated with the completeness of information provided in adverse drug reaction reports of physicians, pharmacists and consumers from Germany
Source: Sci Rep. 2025 Jul 3;15:23751. doi: 10.1038/s41598-025-07973-9 (PMC12229551; doi:10.1038/s41598-025-07973-9)
Supplement: Supplementary file 1 — Supplementary Information 1. [file 41598_2025_7973_MOESM1_ESM.docx]

Supplement 1) Detailed description of the adapted vigiGrade completeness score.

| **Information** | **Description** | **Penalty vigiGrade completeness score^1^** | **Penalty Vigi4Eudra-score** |
| --- | --- | --- | --- |
| Time to onset | Time span between drug intake and ADR occurrence | 50% imprecise or no information 30% uncertainty exceeds 1 month 10% otherwise | 50% (adapted)* |
| Indication | Indication of drug therapy | 30% | 30% |
| Outcome | Outcome of ADR (e.g. recovered, recovering, fatal [..]) | 30% | 30% |
| Sex | Sex of the patient | 30% | 30% |
| Age | Age of the patient | 30% no information 10% if only age group is specified | 30% (adapted)* |
| Dose | Dose of drug therapy | 10% | 10% |
| Country | Country of origin of the ADR report | 10% | 10% |
| Primary reporter | Qualification of the person who reported the case (e.g. physician, pharmacist, consumer) | 10% | 10% |
| Report type | Type of the report (e.g. spontaneous report) | 10% | 10% |
| Comments | Any unstructured free text information | 10% | 10% |

Table from Bergvall et al.^1^, was modified.

*Two adaptations had been made to the original vigiGrade completeness score of Bergvall et al.^1^ developed for the use in vigiBase to meet the structure of the ADR reports from EudraVigilance. For our analysis an extracted line listing of our dataset from EudraVigilance was used. A line listing is a tabular view of the dataset. In this line listing the time to onset could either be determined by calculating the time span between the start date of drug therapy and the start date of the ADR or was classified as not available. Uncertainties like in the original vigiGrade completeness score could not be considered, since exact information regarding the start date of drug therapy and the ADR are either included in the line listing or not. Since the line listing also did not include the category of age groups of the patients only the exact age of the patient could be considered which was either reported or not.

Formula for the calculation:

$$vigiGrade completeness score=\prod_{i=1}^{10} \left( 1-{Penalty}_{i} \right)=\left( 1-P_{1} \right)\ldots\left( 1-P_{10} \right)$$

Formula as published by Bergvall et al.^1^

Example: Regarding the 10 criteria considered in the vigiGrade completeness score, in one ADR report only the information regarding the sex of the patient and the indication of drug therapy are missing.

Table of penalties:

| **Information** | **Description** | **Penalty Vigi4Eudra-score** |
| --- | --- | --- |
| Time to onset | Time span between drug intake and ADR occurrence | No penalty |
| Indication | Indication of drug therapy | 30% |
| Outcome | Outcome of ADR (e.g. recovered, recovering, fatal [..]) | No penalty |
| Sex | Sex of the patient | 30% |
| Age | Age of the patient | No penalty |
| Dose | Dose of drug therapy | No penalty |
| Country | Country of origin of the ADR report | No penalty |
| Primary reporter | Qualification of the person who reported the case (e.g. physician, pharmacist, consumer) | No penalty |
| Report type | Type of the report (e.g. spontaneous report) | No penalty |
| Comments | Any unstructured free text information | No penalty |

vigiGrade completeness score = (1-0)^8*(1-0.3)^2 = 0.49

In case of missing information regarding the age of the patient and the indication of drug therapy, the ADR report would achieve a value of 0.49.

[1] Bergvall, T., Norén, G. N., Lindquist, M. vigiGrade: a tool to identify well-documented individual case reports and highlight systematic data quality issues. Drug Saf. 37(1), 65-77 (2014).
