# Supplementary material for: Factors associated with the completeness of information provided in adverse drug reaction reports of physicians, pharmacists and consumers from Germany
Source: Sci Rep. 2025 Jul 3;15:23751. doi: 10.1038/s41598-025-07973-9 (PMC12229551; doi:10.1038/s41598-025-07973-9)
Supplement: Supplementary file 2 — Supplementary Information 2. [file 41598_2025_7973_MOESM2_ESM.docx]

Supplement 2) Detailed analyses of information provided in ADR reports from physicians, pharmacists and consumers.

S2 Table 1) Detailed analyses of information provided in ADR reports referring to the age groups 0-20 years, 21-59 years and 60 years and older.

| Categories | Age group 0-20 years (n= 8,145 reports) | Age group 21-59 years  (n= 65,269 reports) | Age group 60 years and older (n= 62,674 reports) |
| --- | --- | --- | --- |
| Number of ADR-drug combinations | 28,867 | 256,497 | 242,034 |
| Age of the patient | 100.0% (n= 28,867) | 100.0% (n= 256,497) | 100.0% (n= 242,034) |
| Sex of the patient | 97.0% (n= 28,005) | 99.0% (n= 254,051) | 99.0% (n= 239,567) |
| Indication of drug therapy | 68.4% (n= 19,753) | 64.3% (n= 164,814) | 56.8% (n= 137,434) |
| Dose of drug therapy | 55.5% (n= 16,007) | 58.5% (n= 149,958) | 53.0% (n= 128,231) |
| Time to onset | 45.8% (n= 13,233) | 45.7% (n= 117,326) | 32.6% (n= 78,827) |
| Outcome of the ADR | 57.5% (n= 16,603) | 50.1% (n= 151, 489) | 49.8% (n= 120,616) |
| Narrative | 97.1% (n= 28,032) | 96.1% (n= 264,444) | 96.3% (n= 233,073) |

S2 Table 1 shows the number of ADR-drug combinations reported for patients 0-20 years, 21-59 years and ≥ 60 years and the number of ADR-drug combinations in which specific information is reported. The calculated percentage shares refer to the total number of ADR-drug combinations.

S2 Table 2) Detailed analyses of information provided in ADR reports referring to females, males and sex not specified.

| Categories | Females  (n= 144,535 reports) | Males  (n= 81,145 reports) | Not specified  (n= 7,836 reports) |
| --- | --- | --- | --- |
| Number of ADR-drug combinations | 499,375 | 276,645 | 21,923 |
| Age of the patient | 66.9% (n= 334,163) | 67.4% (n= 186,500) | 26.2% (n= 5,741) |
| Sex of the patient | 100.0% (n= 499,375) | 100.0% (n= 276,645) | 100.0% (n= 21,923) |
| Indication of drug therapy | 56.3% (n= 281,204) | 55.3% (n= 152,981) | 50.0% (n= 10,962) |
| Dose of drug therapy | 53.2% (n= 265,546) | 51.3% (n= 141,839) | 35.9% (n= 7,871) |
| Time to onset | 37.1% (n= 185,336) | 31.2% (n= 86,243) | 18.2% (n= 3,980) |
| Outcome of the ADR | 50.1% (n= 250,061) | 46.4% (n= 128,240) | 32.0% (n= 7,011) |
| Narrative | 96.9% (n= 483,639) | 97.1% (n= 268,562) | 98.6% (n= 21,612) |

S2 Table 2 shows the number of ADR-drug combinations reported for females, males and unknown sex and the number of ADR-drug combinations in which specific information is reported. The calculated percentage shares refer to the total number of ADR-drug combinations.

S2 Table 3) Detailed analyses of information provided in ADR reports received in 2018, 2019, 2020 and 2021 from pharmacists.

| Categories | 2018  (n= 14,036 reports) | 2019  (n= 12,472 reports) | 2020  (n= 8,759 reports) | 2021  (n= 7,129 reports) |
| --- | --- | --- | --- | --- |
| Number of ADR-drug combinations | 36,727 | 33,397 | 27,680 | 25,305 |
| Age of the patient | 67.5% (n= 24,824) | 67.3% (n= 22,481) | 74.7% (n= 20,684) | 76.4% (n= 19,333) |
| Sex of the patient | 96.9% (n= 35,592) | 97.3% (n= 32,499) | 98.3% (n= 27,231) | 98.7% (n= 24,967) |
| Indication of drug therapy | 45.2% (n= 16,611) | 50.5% (n= 16,879) | 46.3% (n= 12,811) | 37.1% (n= 9,387) |
| Dose of drug therapy | 41.2% (n= 15,147) | 47.0% (n= 15,695) | 45.6% (n= 12,626) | 37.3% (n= 9,430) |
| Time to onset | 27.3% (n= 10,028) | 32.2% (n= 10,766) | 31.3% (n= 8,676) | 23.6% (n= 5,970) |
| Outcome of the ADR | 42.7% (n= 15,679) | 48.1% (n= 16,066) | 44.8% (n= 12,399) | 38.2% (n= 9,670) |
| Narrative | 95.7% (n= 35,134) | 95.3% (n= 31,811) | 95.2% (n= 26,351) | 97.4% (n= 24,654) |

S2 Table 3 shows the number of ADR-drug combinations reported in 2018, 2019, 2020 and 2021 and the number of ADR-drug combinations in which specific information is reported. The calculated percentage shares refer to the total number of ADR-drug combinations.

S2Table 4) Detailed analyses of information provided in ADR reports received in 2018, 2019, 2020 and 2021 from consumers.

| Categories | 2018  (n= 26,208 reports) | 2019  (n= 30,858 reports) | 2020  (n= 36,325 reports) | 2021  (n= 27,753 reports) |
| --- | --- | --- | --- | --- |
| Number of ADR-drug combinations | 98,044 | 113,192 | 121,600 | 93,872 |
| Age of the patient | 57.4% (n= 56,286) | 58.9% (n= 66,677) | 60.6% (n= 69,864) | 60.2% (n= 56,555) |
| Sex of the patient | 97.6% (n= 95,730) | 97.0% (n= 109,810) | 98.2% (n= 119,415) | 98.2% (n= 92,215) |
| Indication of drug therapy | 54.1% (n= 53,083) | 51.4% (n= 58,162) | 51.9% (n= 63,052) | 57.8% (n= 54,247) |
| Dose of drug therapy | 52.0% (n= 50,969) | 53.3% (n= 60,379) | 57.5% (n= 69,864) | 55.7% (n= 52,276) |
| Time to onset | 26.3% (n= 25,751) | 34.4% (n= 38,989) | 47.6% (n= 57,940) | 45.0% (n= 42,268) |
| Outcome of the ADR | 43.3% (n= 42,472) | 46.6% (n= 52,751) | 51.8% (n= 63,939) | 58.1% (n= 54,496) |
| Narrative | 97.7% (n= 95,759) | 97.9% (n= 110,789) | 98.0% (n= 119,186) | 97.6% (n= 91,605) |

S2 Table 4 shows the number of ADR-drug combinations reported in 2018, 2019, 2020 and 2021 and the number of ADR-drug combinations in which specific information is reported. The calculated percentage shares refer to the total number of ADR-drug combinations.
