# Supplementary material for: Factors associated with the completeness of information provided in adverse drug reaction reports of physicians, pharmacists and consumers from Germany
Source: Sci Rep. 2025 Jul 3;15:23751. doi: 10.1038/s41598-025-07973-9 (PMC12229551; doi:10.1038/s41598-025-07973-9)
Supplement: Supplementary file 3 — Supplementary Information 3. [file 41598_2025_7973_MOESM3_ESM.docx]

Supplement 3) Boxplots showing the mean and the median values of the vigiGrade completeness scores concerning serious ADR reports and the seriousness criteria death, hospitalization, life-threatening and disabling.

S3 Figure 1) Association of seriousness of the ADR report on the completeness of ADR reports.


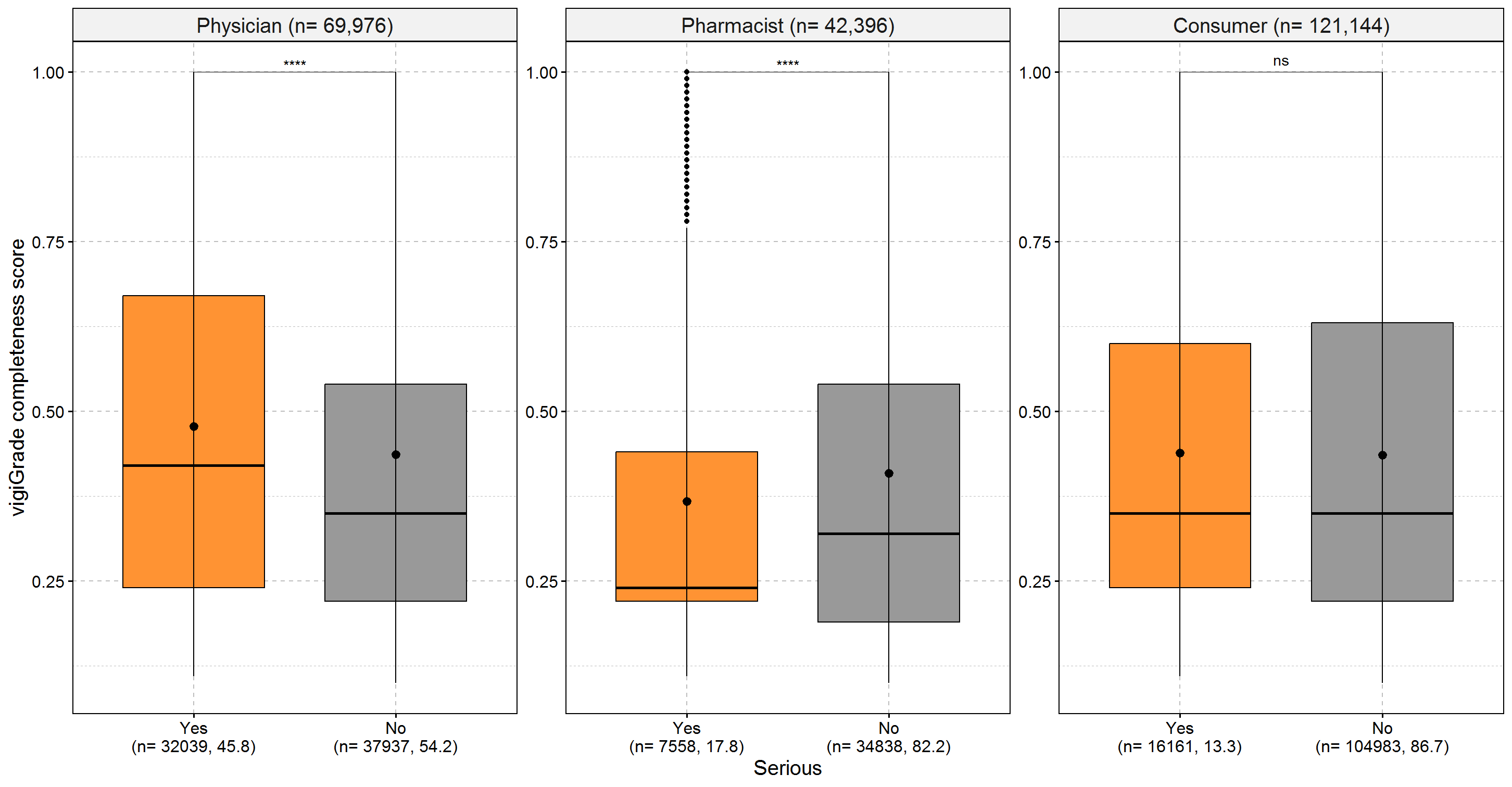


p-values coded as: 1-0.05 ‘ns’; >0.05-0.01 ‘*’; >0.01-0.001 ‘**’; >0.001-0.0001 ‘***’; >0.0001-0 ‘****’

S3 Figure 1) shows the boxplots of the vigiGrade completeness scores with the corresponding mean and median values depending on the seriousness of ADR reports from physicians, pharmacists and consumers. An unpaired t-test with Holm’s correction for multiple testing was performed to analyse differences between the mean values of the vigiGrade completeness scores of the analysed categories.

S3 Figure 2) Association of seriousness criterion death on the completeness of ADR reports.
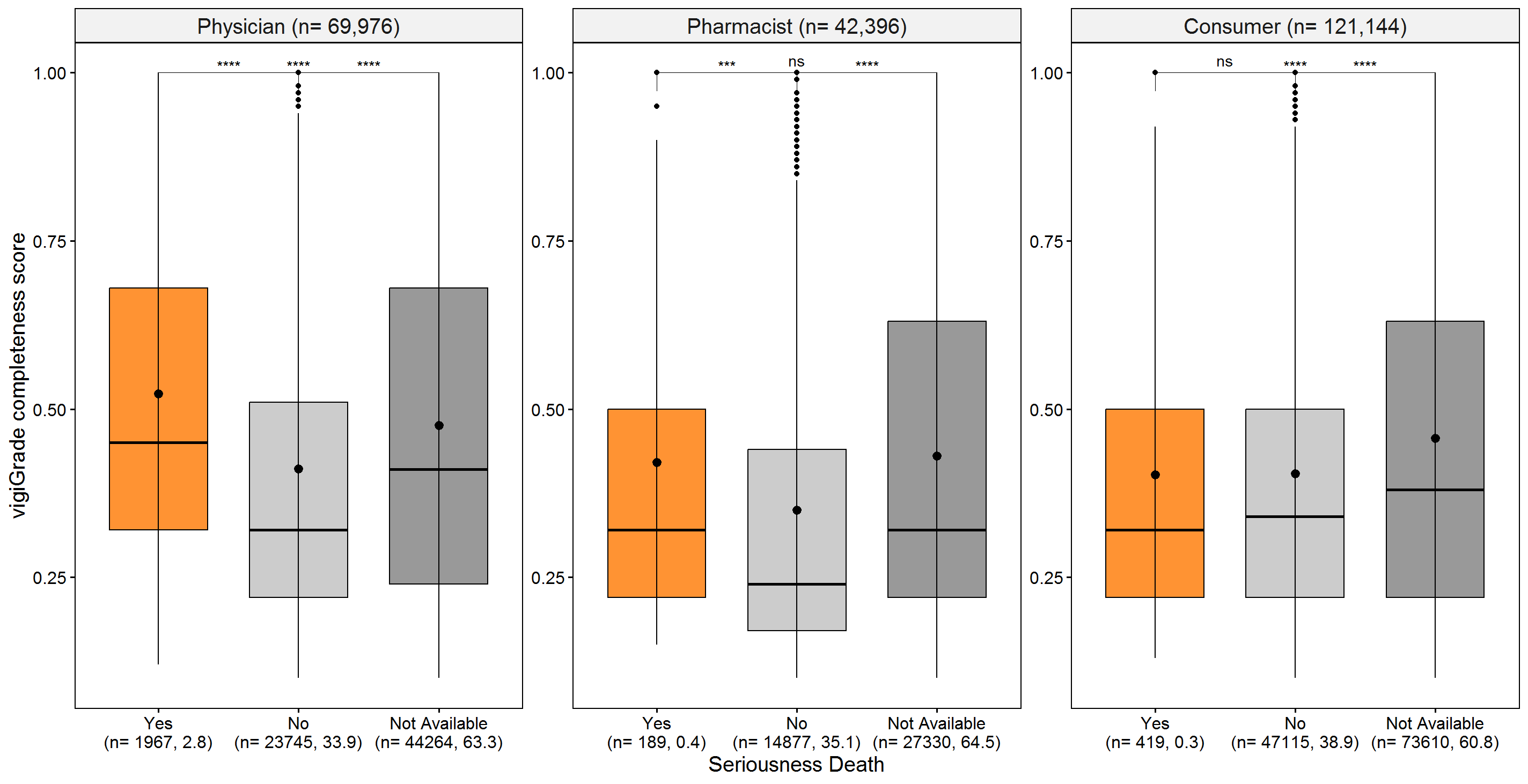


p values coded as: 1-0.05 ‘ns’; >0.05-0.01 ‘*’; >0.01-0.001 ‘**’; >0.001-0.0001 ‘***’; >0.0001-0 ‘****’

S3 Figure 2) shows the boxplots of the vigiGrade completeness scores with the corresponding mean and median values depending on the seriousness criterion death of ADR reports from physicians, pharmacists and consumers. An unpaired t-test with Holm’s correction for multiple testing was performed to analyse differences between the mean values of the vigiGrade completeness scores of the analysed categories.

S3 Figure 3) Association of seriousness criterion hospitalisation on the completeness of ADR reports.


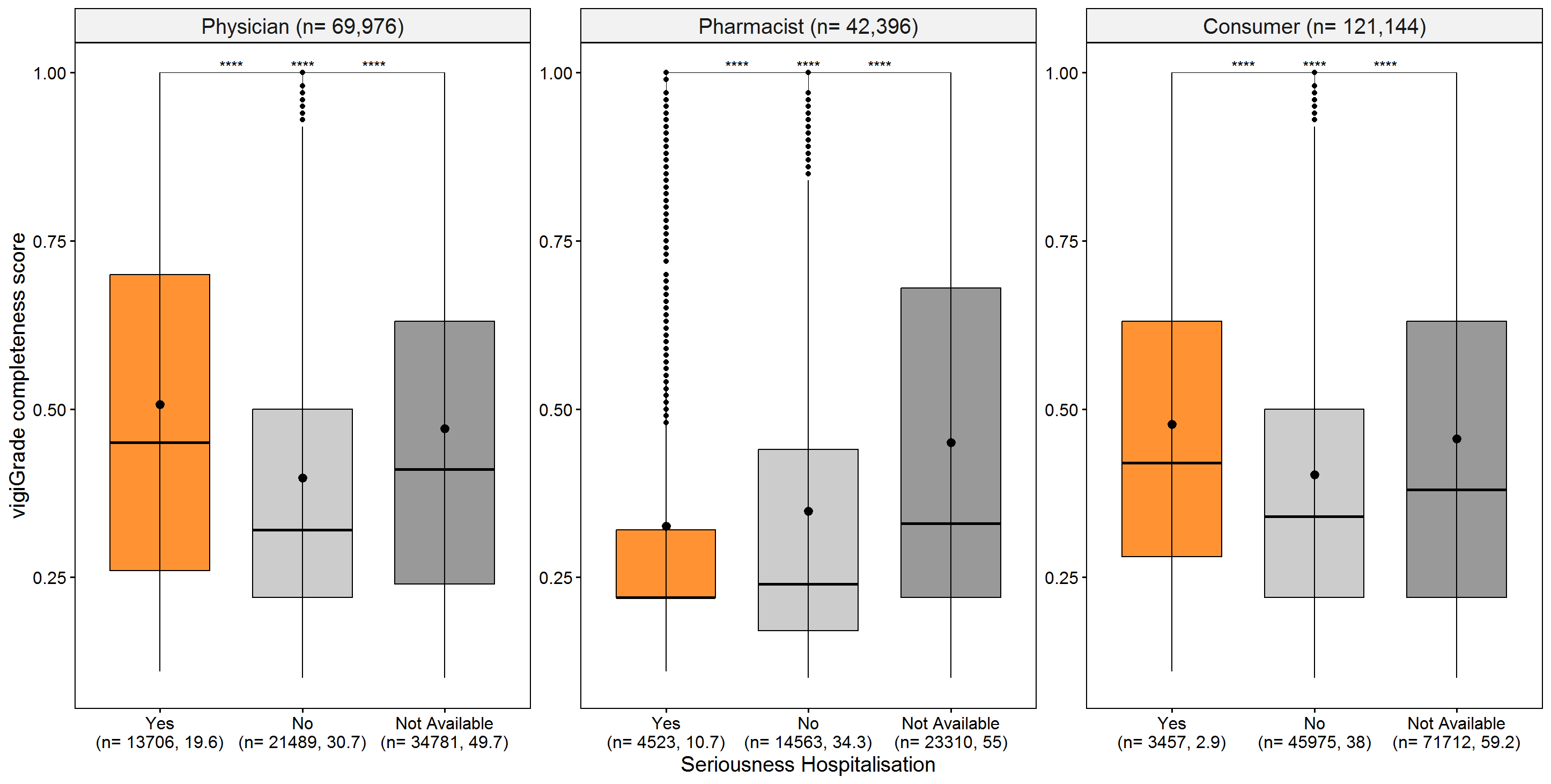


p values coded as: 1-0.05 ‘ns’; >0.05-0.01 ‘*’; >0.01-0.001 ‘**’; >0.001-0.0001 ‘***’; >0.0001-0 ‘****’

S3 Figure 3) shows the boxplots of the vigiGrade completeness scores with the corresponding mean and median values depending on the seriousness criterion hospitalization of ADR reports from physicians, pharmacists and consumers. An unpaired t-test with Holm’s correction for multiple testing was performed to analyse differences between the mean values of the vigiGrade completeness scores of the analysed categories.

S3 Figure 4) Association of seriousness criterion life-threatening on the completeness of ADR reports.


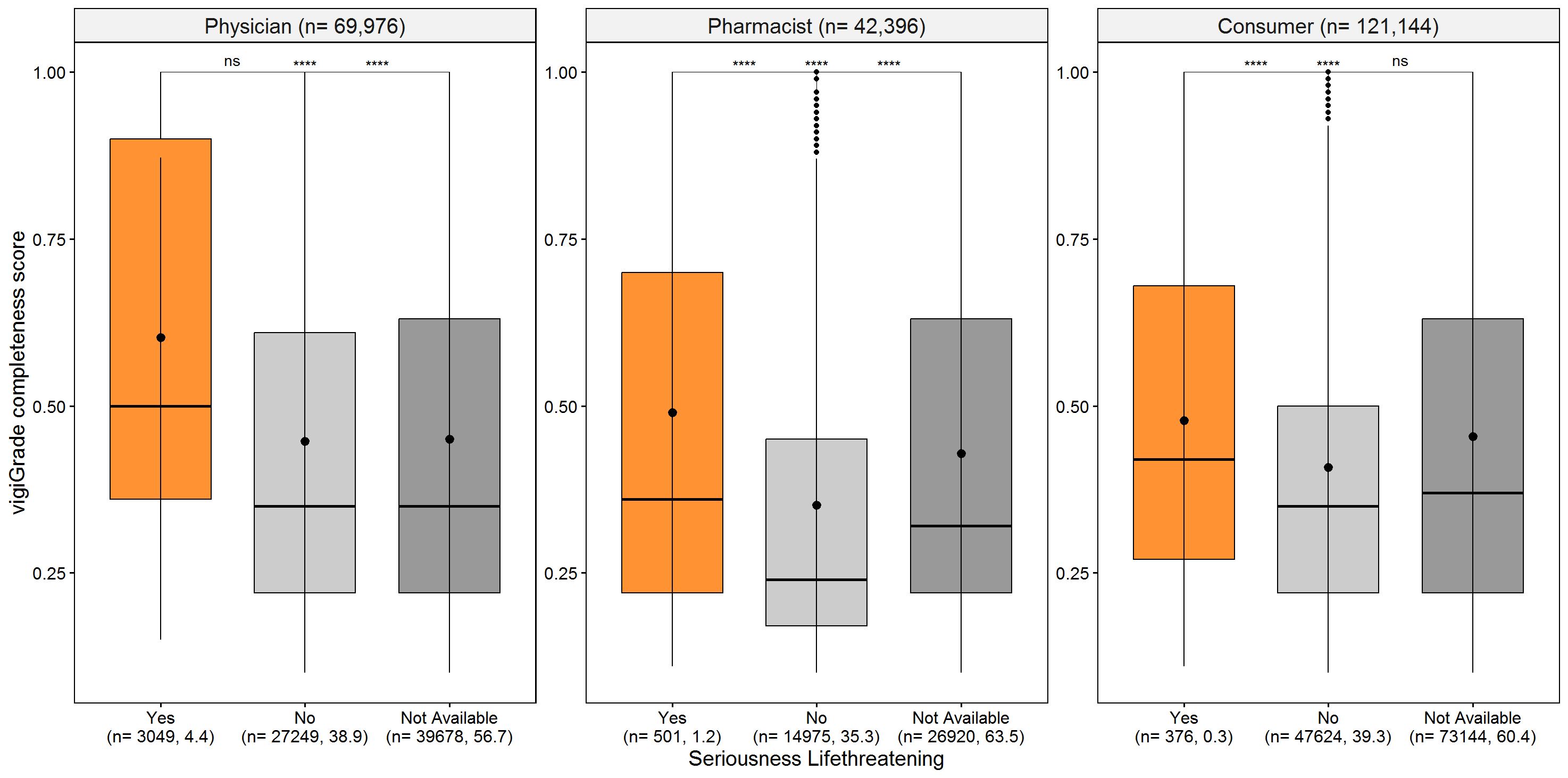


p values coded as: 1-0.05 ‘ns’; >0.05-0.01 ‘*’; >0.01-0.001 ‘**’; >0.001-0.0001 ‘***’; >0.0001-0 ‘****’

S3 Figure 4) shows the boxplots of the vigiGrade completeness scores with the corresponding mean and median values depending on the seriousness criterion life-threatening of ADR reports from physicians, pharmacists and consumers. An unpaired t-test with Holm’s correction for multiple testing was performed to analyse differences between the mean values of the vigiGrade completeness scores of the analysed categories.

S3 Figure 5) Association of seriousness criterion disabling on the completeness of ADR reports.


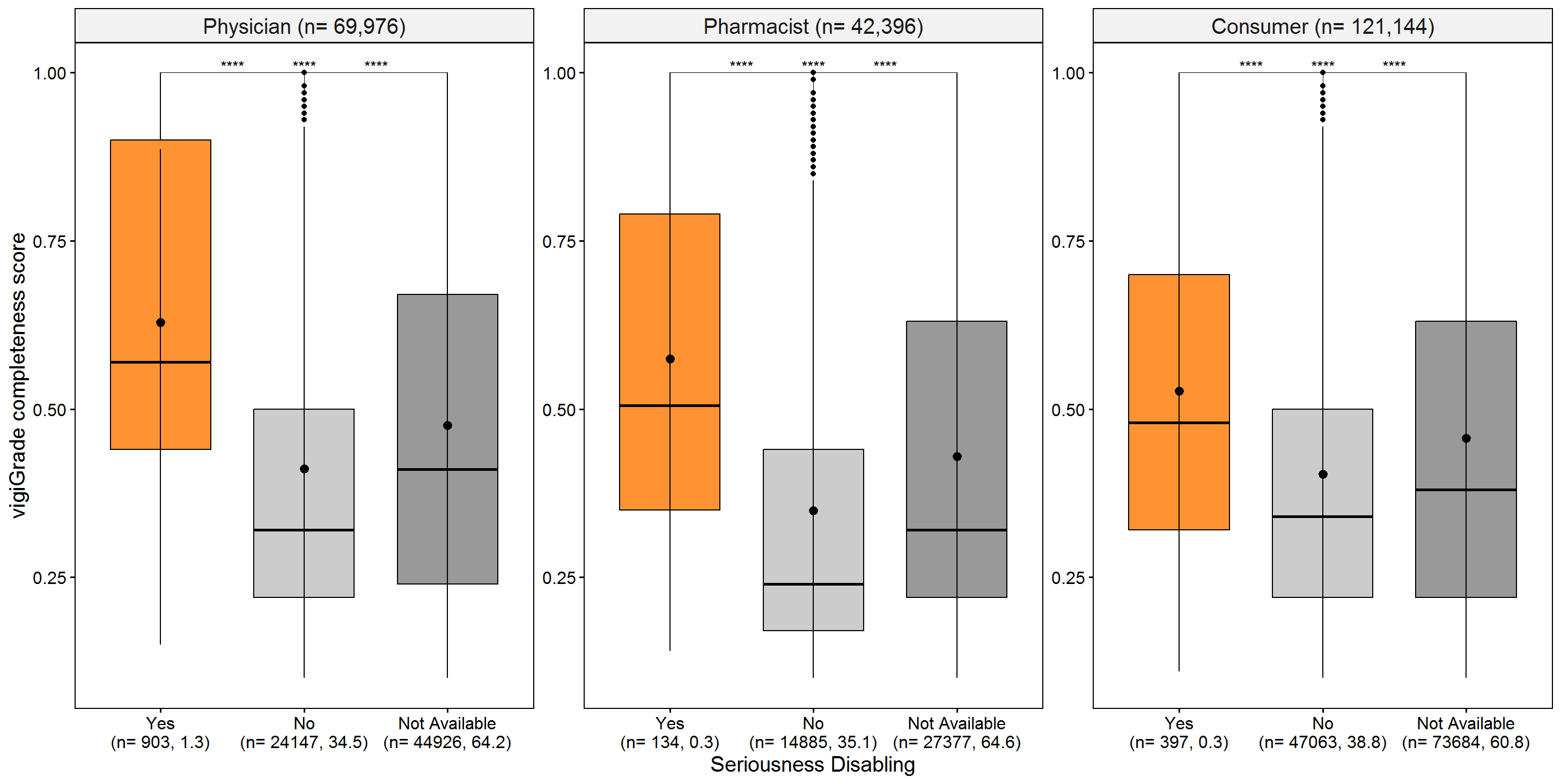


p values coded as: 1-0.05 ‘ns’; >0.05-0.01 ‘*’; >0.01-0.001 ‘**’; >0.001-0.0001 ‘***’; >0.0001-0 ‘****’

S3 Figure 5) shows the boxplots of the vigiGrade completeness scores with the corresponding mean and median values depending on the seriousness criterion disabling of ADR reports from physicians, pharmacists and consumers. An unpaired t-test with Holm’s correction for multiple testing was performed to analyse differences between the mean values of the vigiGrade completeness scores of the analysed categories.
