# Supplementary material for: Factors associated with the completeness of information provided in adverse drug reaction reports of physicians, pharmacists and consumers from Germany
Source: Sci Rep. 2025 Jul 3;15:23751. doi: 10.1038/s41598-025-07973-9 (PMC12229551; doi:10.1038/s41598-025-07973-9)
Supplement: Supplementary file 4 — Supplementary Information 4. [file 41598_2025_7973_MOESM4_ESM.docx]

Supplement 4) Association of the number of histories provided in each ADR report with the completeness of the ADR report.

S4 Figure 1) Association of the number of histories provided in each ADR report with the completeness of the ADR report.


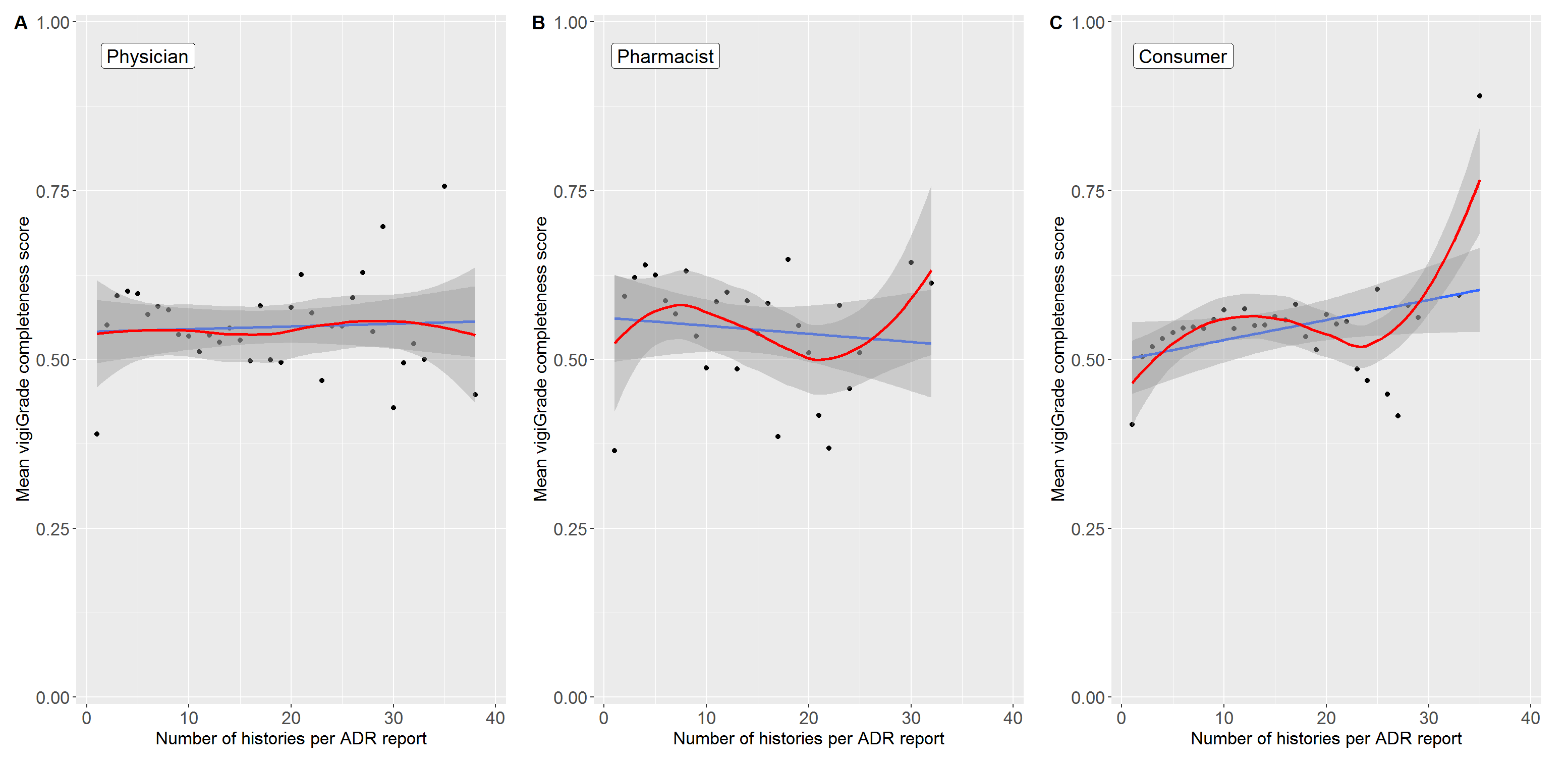


S4 Figure 1 shows the mean values of the vigiGrade completeness scores depending on the number of histories provided in ADR reports from physicians, pharmacists and consumers. The blue line represents the linear regression line and the red line the loess regression line. For smoothing of the latter, spans of 0.95, 0.90 and 0.90 were set for ADR reports from physicians, pharmacists and consumers, respectively. The gray borders represent the 95.0% confidence intervals.
