# Supplementary material for: Factors associated with the completeness of information provided in adverse drug reaction reports of physicians, pharmacists and consumers from Germany
Source: Sci Rep. 2025 Jul 3;15:23751. doi: 10.1038/s41598-025-07973-9 (PMC12229551; doi:10.1038/s41598-025-07973-9)
Supplement: Supplementary file 5 — Supplementary Information 5. [file 41598_2025_7973_MOESM5_ESM.docx]

Supplement 5) Association of the number of ADRs reported in each ADR report with the completeness of the ADR report.

S5 Figure 1) Association of the number of ADRs reported in each ADR report with the completeness of the ADR report.


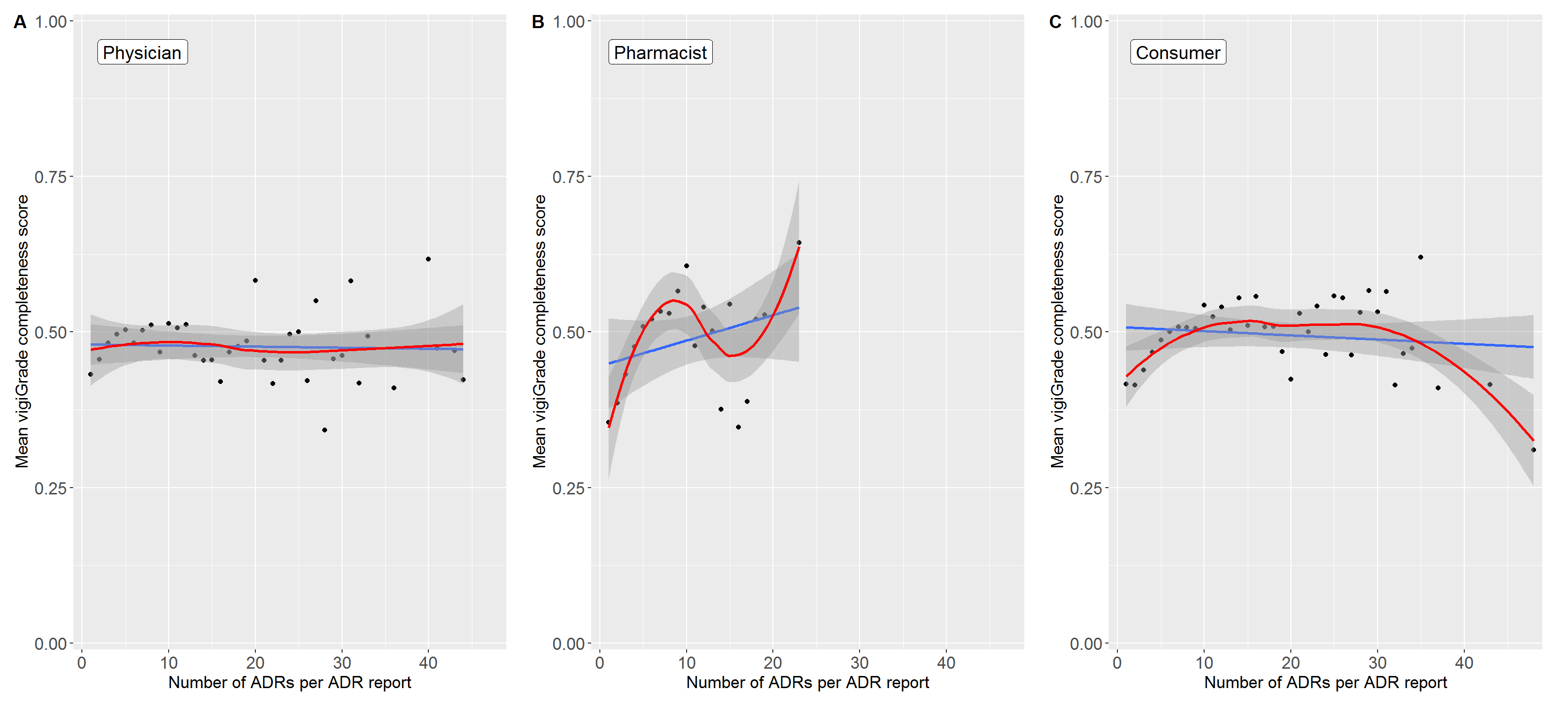


S5 Figure 1 shows the mean values of the vigiGrade completeness scores depending on the number of ADRs per ADR report from physicians, pharmacists and consumers. The blue line represents the regression line and the red line the loess regression line. For smoothing of the latter, a span of 0.90 was set for ADR reports from all three reporter types. The gray borders represent the 95.0% confidence intervals.
