# Supplementary material for: Factors associated with the completeness of information provided in adverse drug reaction reports of physicians, pharmacists and consumers from Germany
Source: Sci Rep. 2025 Jul 3;15:23751. doi: 10.1038/s41598-025-07973-9 (PMC12229551; doi:10.1038/s41598-025-07973-9)
Supplement: Supplementary file 6 — Supplementary Information 6. [file 41598_2025_7973_MOESM6_ESM.docx]

Supplement 6) Analyses of the influence of the type of ADR on the SOC level on the completeness of ADR reports from physicians, pharmacists and consumers.

S6 Figure 1) Association of the type of ADR on the SOC level on the completeness of ADR reports from physicians.


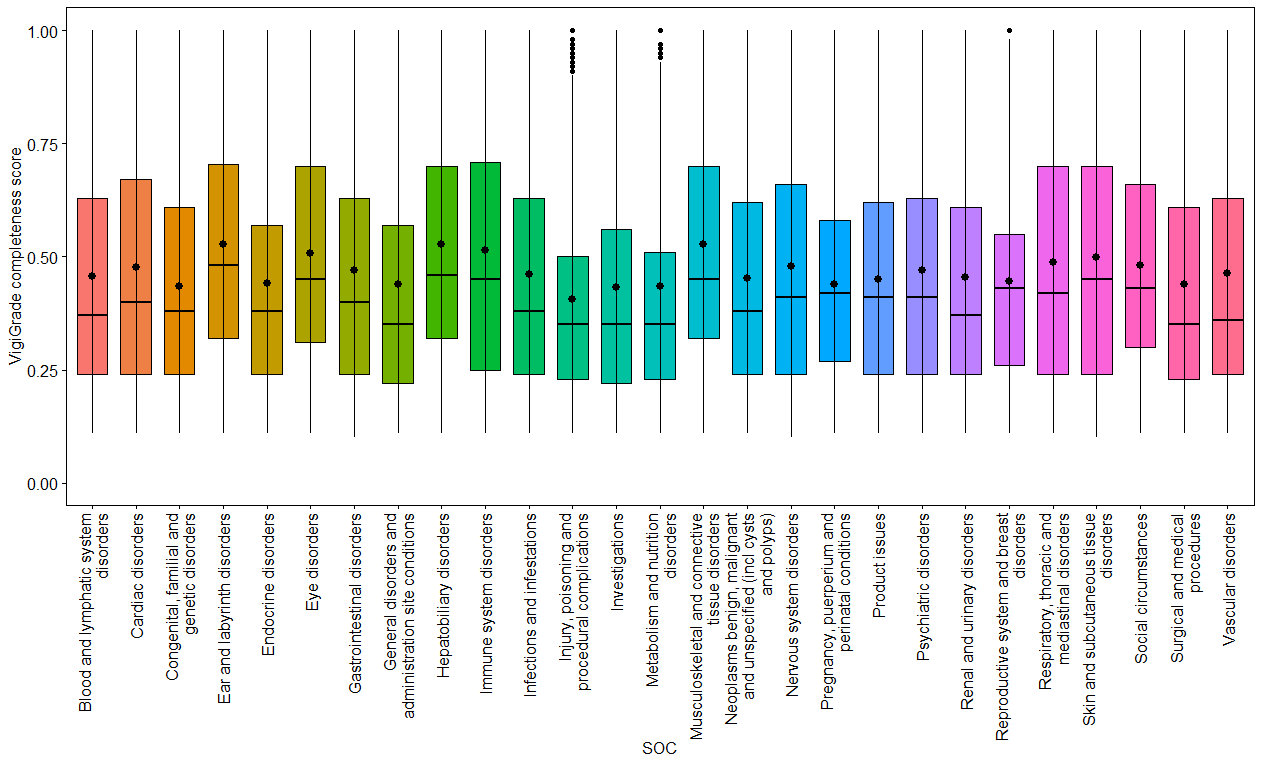


S6 Figure 2) Association of the type of ADR on the SOC level on the completeness of ADR reports from pharmacists.


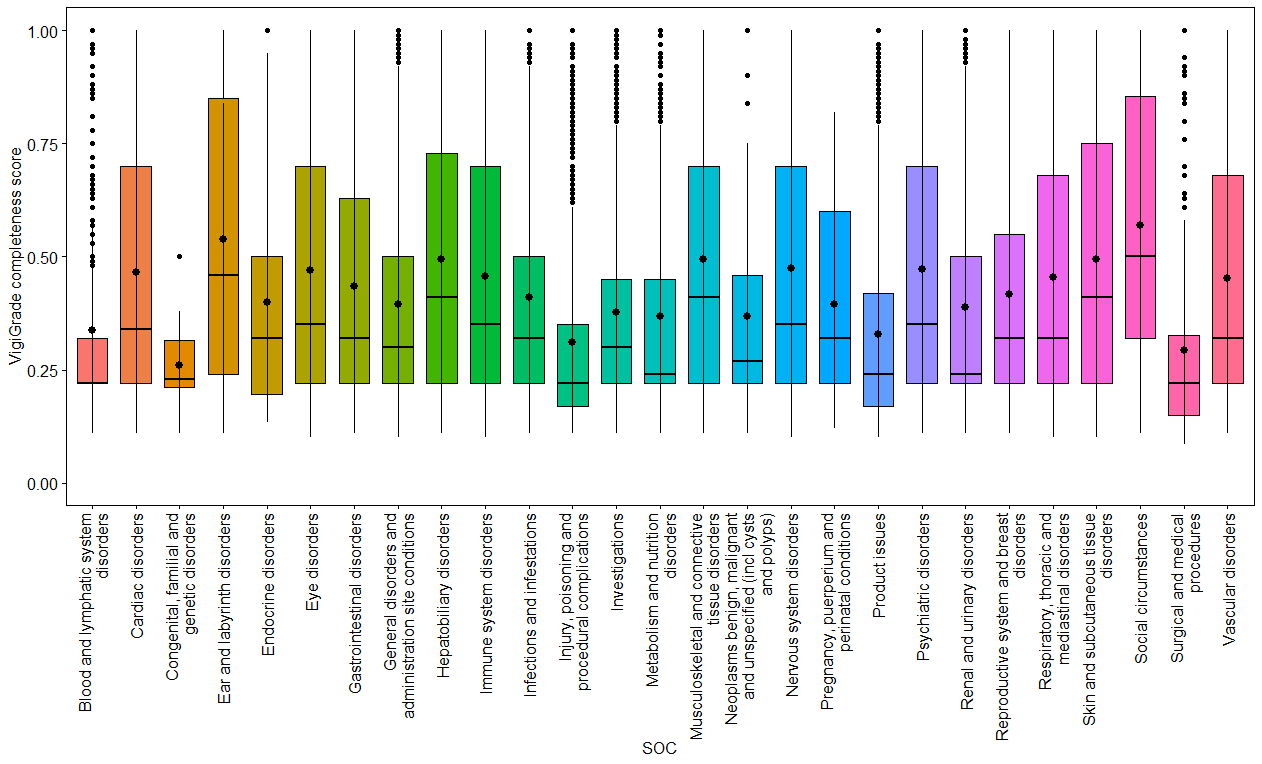


S6 Figure 3) Association of the type of ADR on the SOC level on the completeness of ADR reports from consumers.


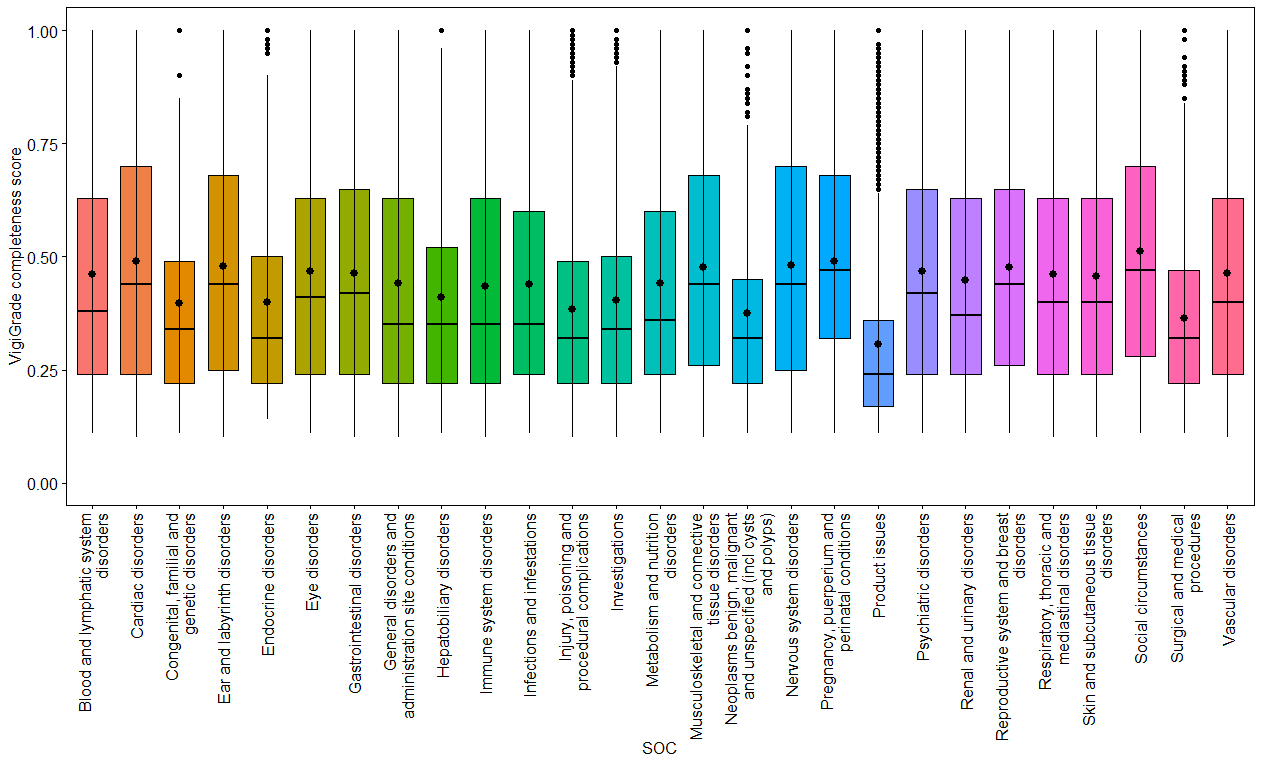


S6 Table 1) Association of the type of ADR on the SOC level on the completeness of ADR reports from physicians, pharmacists and consumers.

| SOC level | Physician:  Mean VigiGrade completeness scores (+/-sd) | Physician:  Median VigiGrade completeness scores [IQR] | Pharmacist:  Mean VigiGrade completeness scores (+/-sd) | Pharmacist:  Median VigiGrade completeness scores [IQR] | Consumer:  Mean VigiGrade completeness scores (+/-sd) | Consumer:  Median VigiGrade completeness scores [IQR] |
| --- | --- | --- | --- | --- | --- | --- |
| Blood and lymphatic system disorders | 0.46 (+/-0.26) | 0.37 [0.24-0.63] | 0.34 (+/-0.22) | 0.22 [0.22-0.32] | 0.46 (+/-0.25) | 0.38 [0.24-0.63] |
| Cardiac disorders | 0.48 (+/-0.27) | 0.40 [0.24-0.67] | 0.47 (+/-0.29) | 0.34 [0.22-0.70] | 0.49 (+/-0.26) | 0.44 [0.24-0.70] |
| Congenital, familial and genetic disorders | 0.43 (+/-0.23) | 0.38 [0.24-0.61] | 0.26 (+/-0.09) | 0.23 [0.21-0.32] | 0.40 (+/-0.22) | 0.34 [0.22-0.49] |
| Ear and labyrinth disorders | 0.53 (+/-0.28) | 0.48 [0.32-0.71] | 0.54 (+/-0.30) | 0.46 [0.24-0.85] | 0.48 (+/-0.25) | 0.44 [0.25-0.68] |
| Endocrine disorders | 0.44 (+/- 0.23) | 0.38 [0.24-0.57] | 0.40 (+/-0.27) | 0.32 [0.20-0.50] | 0.40 (+/-0.22) | 0.32 [0.22-0.50] |
| Eye disorders | 0.51 (+/- 0.26) | 0.45 [0.31-0.70] | 0.47 (+/-0.47] | 0.35 [0.22-0.70] | 0.47 (+/-0.26) | 0.41 [0.24-0.63] |
| Gastrointestinal disorders | 0.47 (+/-0.27) | 0.40 [0.24-0.63] | 0.44 (+/-0.29) | 0.32 [0.22-0.63] | 0.46 (+/-0.26) | 0.42 [0.24-0.65] |
| General disorders and administration site conditions | 0.44 (+/-0.25) | 0.35 [0.22-0.57] | 0.40 (+/-0.27) | 0.30 [0.22-0.50] | 0.44 (+/-0.26) | 0.35 [0.22-0.63] |
| Hepatobiliary disorders | 0.53 (+/-0.27) | 0.46 [0.32-0.70] | 0.49 (+/-0.30) | 0.41 [0.22-0.73] | 0.41 (+/-0.23) | 0.35 [0.22-0.52] |
| Immune system disorders | 0.51 (+/-0.29) | 0.45 [0.25-0.71] | 0.46 (+/-0.30) | 0.35 [0.22-0.70] | 0.43 (+/-0.27) | 0.35 [0.22-0.63] |
| Infections and infestations | 0.46 (+/-0.26) | 0.38 [0.24-0.63] | 0.41 (+/-0.27) | 0.32 [0.22-0.50] | 0.44 (+/-0.25) | 0.35 [0.24-0.60] |
| Injury, poisoning and procedural complications | 0.41 (+/-0.22) | 0.35 [0.23-0.50] | 0.31 (+/-0.20) | 0.22 [0.17-0.35] | 0.38 (+/-0.21) | 0.32 [0.22-0.49] |
| Investigations | 0.43 (+/- 0.25) | 0.35 [0.22-0.56] | 0.38 (+/-0.25) | 0.30 [0.22-0.50] | 0.40 (+/-0.23) | 0.34 [0.22-0.50] |
| Metabolism and nutrition disorders | 0.44 (+/-0.24) | 0.35 [0.23-0.51] | 0.37 (+/-0.25) | 0.24 [0.22-0.45] | 0.44 (+/-0.24) | 0.36 [0.24-0.60] |
| Musculoskeletal and connective tissue disorders | 0.53 (+/-0.27) | 0.45 [0.32-0.70] | 0.49 (+/-0.30) | 0.41 [0.22-0.70] | 0.48 (+/-0.25) | 0.44 [0.26-0.68] |
| Neoplasms benign, malignant and  unspecified (incl cysts and polyps) | 0.45 (+/-0.24) | 0.38 [0.24-0.62] | 0.37 (+/-0.24) | 0.27 [0.22-0.46] | 0.37 (+/-0.21) | 0.32 [0.22-0.45] |
| Nervous system disorders | 0.48 (+/-0.27) | 0.41 [0.24-0.66] | 0.47 (+/-0.29) | 0.35 [0.22-0.70] | 0.48 (+/-0.26) | 0.44 [0.25-0.70] |
| Pregnancy, puerperium and perinatal conditions | 0.44 (+/-0.20) | 0.42 [0.27-0.58] | 0.39 (+/-0.22) | 0.32 [0.22-0.60] | 0.49 (+/-0.22) | 0.47 [0.32-0.68] |
| Product issues | 0.45 (+/-0.24) | 0.41 [0.24-0.62] | 0.33 (+/-0.22) | 0.24 [0.17-0.42] | 0.31 (+/-0.19) | 0.24 [0.17-0.36] |
| Psychiatric disorders | 0.47 (+/-0.26) | 0.41 [0.24-0.63] | 0.47 (+/-0.29) | 0.35 [0.22-0.70] | 0.47 (+/-0.25) | 0.42 [0.24-0.65] |
| Renal and urinary disorders | 0.46 (+/-0.25) | 0.37 [0.24-0.61] | 0.39 (+/-0.25) | 0.24 [0.22-0.50] | 0.45 (+/-0.25) | 0.37 [0.24-0.63] |
| Reproductive system and breast disorders | 0.45 (+/-0.23) | 0.43 [0.26-0.55] | 0.42 (+/-0.27) | 0.32 [0.22-0.55] | 0.48 (+/-0-25) | 0.44 [0.26-0.65] |
| Respiratory, thoracic and mediastinal disorders | 0.49 (+/-0.27) | 0.42 [0.24-0.70] | 0.45 (+/-0.29) | 0.32 [0.22-0.68] | 0.46 (+/-0.26) | 0.40 [0.24-0.63] |
| Skin and subcutaneous tissue disorders | 0.50 (+/-0.28) | 0.45 [0.24-0.70] | 0.49 (+/-0.30) | 0.41 [0.22-0.75] | 0.46 (+/-0.26) | 0.40 [0.24-0.63] |
| Social circumstances | 0.48 (+/-0.24) | 0.43 [0.30-0.66] | 0.57 (+/-0.28) | 0.50 [0.32-0.86] | 0.51 (+/-0.26) | 0.47 [0.27-0.70] |
| Surgical and medical procedures | 0.44 (+/-0.25) | 0.35 [0.23-0.61] | 0.29 (+/-0.21) | 0.22 [0.15-0.33] | 0.36 (+/-0.21) | 0.32 [0.22-0.47] |
| Vascular disorders | 0.46 (+/-0.26) | 0.36 [0.24-0.63] | 0.45 (+/-0.29) | 0.32 [0.22-0.68] | 0.46 (+/-0.25) | 0.40 [0.24-0.63] |
