# Supplementary material for: Factors associated with the completeness of information provided in adverse drug reaction reports of physicians, pharmacists and consumers from Germany
Source: Sci Rep. 2025 Jul 3;15:23751. doi: 10.1038/s41598-025-07973-9 (PMC12229551; doi:10.1038/s41598-025-07973-9)
Supplement: Supplementary file 7 — Supplementary Information 7. [file 41598_2025_7973_MOESM7_ESM.docx]

Supplement 7) The mean values of the vigiGrade completeness scores of the 25 most frequently reported ADR-drug combinations per reporter type.

S7 Table 1) The 25 most frequently reported ADR-drug combinations in ADR reports from physicians.

| ADR-drug combination | Number of ADR reports | Mean value of the vigiGrade completeness score | Median value of the vigiGrade completeness score |
| --- | --- | --- | --- |
| Myalgia - atorvastatin | 815 | 0.53 | 0.45 |
| Myalgia - rosuvastatin | 689 | 0.58 | 0.50 |
| Myalgia – simvastatin | 579 | 0.49 | 0.45 |
| Drug ineffective - levonorgestrel | 505 | 0.29 | 0.32 |
| Myalgia – ezetimibe | 383 | 0.59 | 0.50 |
| Lymphopenia – dimethyl fumarate | 372 | 0.45 | 0.35 |
| Ovarian hyperstimulation syndrome - follitropin | 361 | 0.51 | 0.45 |
| Device expulsion . levonorgestrel | 326 | 0.53 | 0.49 |
| Myalgia - evolocumab | 298 | 0.55 | 0.50 |
| Myalgia - pravastatin | 285 | 0.48 | 0.45 |
| Ovarian hyperstimulation syndrome - ganirelix | 270 | 0.51 | 0.45 |
| Ectopic pregnancy with contraceptive device - levonorgestrel | 255 | 0.62 | 0.70 |
| Pregnancy with contraceptive device – levonorgestrel | 254 | 0.59 | 0.63 |
| Complication of device insertion - levonorgestrel | 246 | 0.51 | 0.44 |
| Myalgia - fluvastatin | 236 | 0.53 | 0.45 |
| Ovarian hyperstimulation syndrome - choriogonadotropin | 233 | 0.50 | 0.45 |
| Device dislocation - levonorgestrel | 225 | 0.52 | 0.49 |
| Multiple sclerosis relapse - natalizumab | 216 | 0.33 | 0.22 |
| Diarrhoea - nintedanib | 204 | 0.34 | 0.25 |
| Balanoposthitis - empagliflozin | 203 | 0.34 | 0.25 |
| Hypoglycaemia - insulin | 192 | 0.38 | 0.25 |
| Urinary tract infection - empagliflozin | 184 | 0.27 | 0.22 |
| Off label use - botulinium | 182 | 0.33 | 0.32 |
| Cerebral haemorrhage - apixaban | 180 | 0.46 | 0.35 |
| Haematochezia – acetylsalicylic acid | 176 | 0.24 | 0.22 |

S7 Table 1 shows the 25 most frequently reported ADR-drug combinations in ADR reports from physicians and their mean and median values of the vigiGrade completeness score.

S7 Table 2) The 25 most frequently reported ADR-drug combinations in ADR reports from pharmacists.

| ADR-drug combinations | Number of ADR reports | Mean value of the vigiGrade completeness score | Median value of the vigiGrade completeness score |
| --- | --- | --- | --- |
| Blood glucose increased - insulin | 1136 | 0.31 | 0.22 |
| Device breakage - ethinylestradiol | 631 | 0.22 | 0.17 |
| Drug ineffective - insulin | 560 | 0.28 | 0.22 |
| Pruritus - minoxidil | 221 | 0.52 | 0.45 |
| Pain - ethinylestradiol | 172 | 0.25 | 0.17 |
| Application site pruritus - minoxidil | 160 | 0.52 | 0.45 |
| Hypoglycaemia - insulin | 159 | 0.28 | 0.22 |
| Eye irritation -azelastine | 150 | 0.41 | 0.32 |
| No adverse event - leuprorelin | 148 | 0.21 | 0.22 |
| Haematochezia – acetylsalicylic acid | 142 | 0.23 | 0.22 |
| Injection site pain - enoxaparin | 136 | 0.39 | 0.32 |
| Erythema - minoxidil | 134 | 0.54 | 0.50 |
| Blood glucose abnormal - insulin | 128 | 0.27 | 0.22 |
| Application site erythema - rotigotine | 121 | 0.31 | 0.25 |
| Nausea - dry | 115 | 0.63 | 0.50 |
| Application site pruritus - heparin | 111 | 0.60 | 0.50 |
| Dyspnoea - paclitaxel | 105 | 0.56 | 0.50 |
| Liquid product physical issue - insulin | 101 | 0.26 | 0.22 |
| Headache - minoxidil | 99 | 0.57 | 0.45 |
| Application site erythema - heparin | 98 | 0.57 | 0.50 |
| Diarrhoea - pancreatin | 98 | 0.37 | 0.25 |
| Dyspnoea - formoterol | 97 | 0.26 | 0.22 |
| Incorrect dose administered - insulin | 97 | 0.25 | 0.22 |
| Product quality issue - insulin | 97 | 0.30 | 0.22 |
| Application site erythema - buprenorphine | 95 | 0.38 | 0.32 |

S7 Table 2 shows the 25 most frequently reported ADR-drug combinations in ADR reports from pharmacists and their mean and median values of the vigiGrade completeness score.

S7 Table 3) The 25 most frequently reported ADR-drug combinations in ADR reports from consumers.

| ADR-drug combinations | Number of ADR reports | Mean value of the vigiGrade completeness score | Median value of the vigiGrade completeness score |
| --- | --- | --- | --- |
| Blood glucose increased - insulin | 1703 | 0.33 | 0.32 |
| Alopecia - minoxidil | 846 | 0.33 | 0.25 |
| Injection site pain - glatiramer | 802 | 0.66 | 0.70 |
| Flushing – dimethyl fumarate | 710 | 0.56 | 0.50 |
| Product leakage - insulin | 584 | 0.33 | 0.32 |
| Injection site erythema - glatiramer | 495 | 0.66 | 0.70 |
| Injection site induration - glatiramer | 482 | 0.65 | 0.50 |
| Device breakage - ethinylestradiol | 464 | 0.22 | 0.17 |
| Fatigue - levothyroxine | 445 | 0.40 | 0.31 |
| Abdominal pain upper – dimethyl fumarate | 437 | 0.56 | 0.50 |
| Injection site swelling - glatiramer | 418 | 0.65 | 0.70 |
| Dizziness - levothyroxine | 375 | 0.43 | 0.32 |
| Hyperglycaemia - insulin | 372 | 0.41 | 0.32 |
| Headache - levothyroxine | 356 | 0.49 | 0.44 |
| Palpitations - levothyroxine | 348 | 0.45 | 0.35 |
| Diarrhoea – dimethyl fumarate | 338 | 0.56 | 0.49 |
| Off label use - misoprostol | 337 | 0.44 | 0.35 |
| Multiple sclerosis relapse – dimethyl fumarate | 326 | 0.54 | 0.49 |
| Restlessness - levothyroxine | 306 | 0.45 | 0.35 |
| Nausea - levothyroxine | 304 | 0.42 | 0.34 |
| Injection site pruritus - glatiramer | 294 | 0.65 | 0.70 |
| Drug ineffective - insulin | 292 | 0.29 | 0.32 |
| Diarrhoea - metformin | 290 | 0.42 | 0.35 |
| Hyperhidrosis – levothyroxine | 289 | 0.42 | 0.32 |
| Dizziness - valsartan | 287 | 0.33 | 0.25 |

S7 Table 3 shows the 25 most frequently reported ADR-drug combinations in ADR reports from consumers and their mean and median values of the vigiGrade completeness score.
