# Supplementary material for: Factors associated with the completeness of information provided in adverse drug reaction reports of physicians, pharmacists and consumers from Germany
Source: Sci Rep. 2025 Jul 3;15:23751. doi: 10.1038/s41598-025-07973-9 (PMC12229551; doi:10.1038/s41598-025-07973-9)
Supplement: Supplementary file 8 — Supplementary Information 8. [file 41598_2025_7973_MOESM8_ESM.docx]

Supplement 8) Association of the number of words reported in the narrative with the assessment of the causal relationship.

S8 Figure 1) Association of the number of words reported in the narrative with the assessment of the causal relationship.


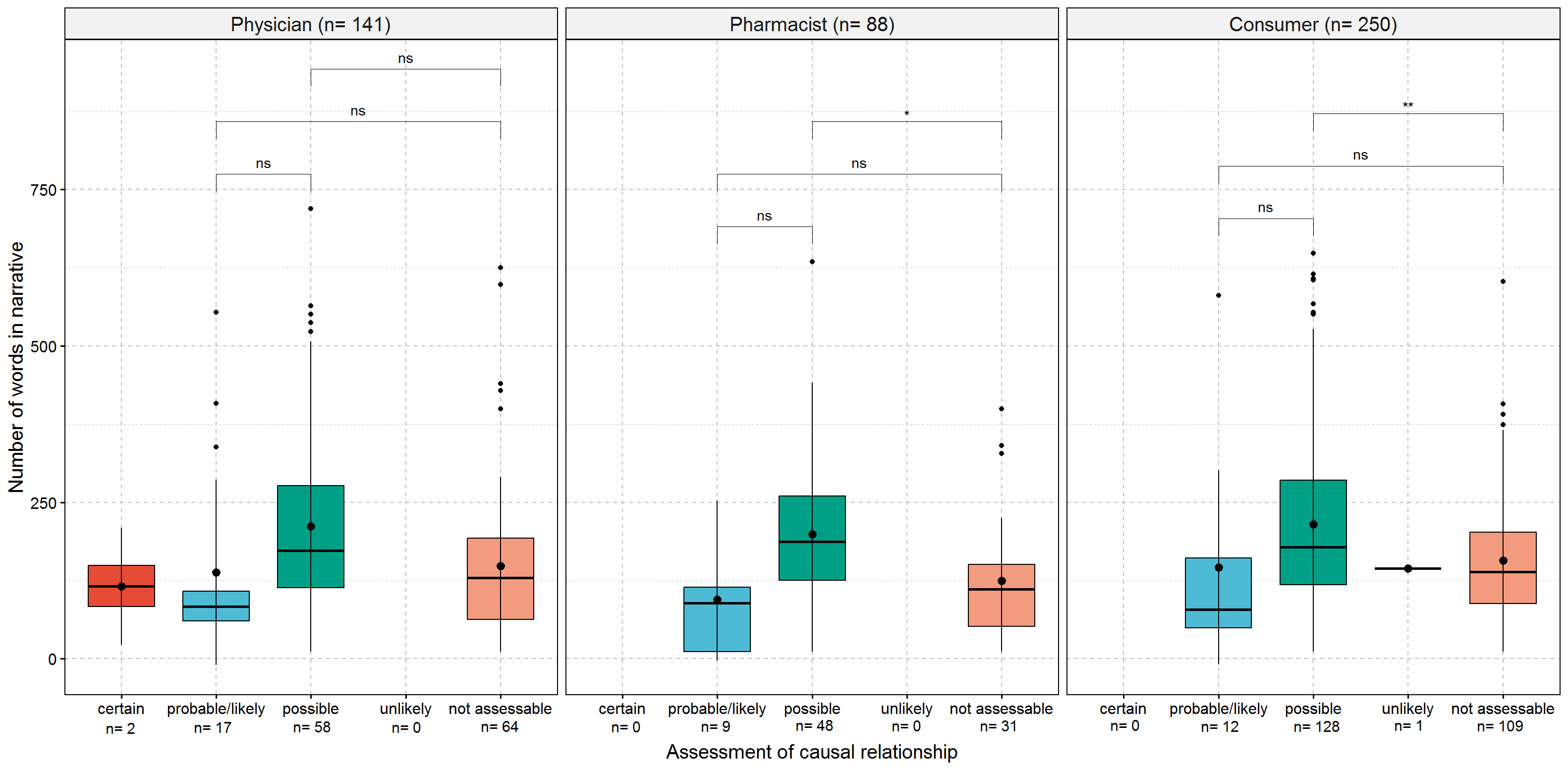


p-values coded as: 1-0.05 ‘ns’; >0.05-0.01 ‘*’; >0.01-0.001 ‘**’; >0.001-0.0001 ‘***’; >0.0001-0 ‘****’

S8 Figure 1 shows the boxplots with the mean and median number of words used in narratives in the ADR reports from physicians, pharmacists and consumers with a certain, probable, possible, unlikely or not assessable causal relationship. An unpaired t-test with Holm’s correction for multiple testing was performed to analyse differences between the mean number of words used in the narratives of the analysed categories. The categories certain and unlikely could not be included in statistical analysis due to their low number of reports.
